# Small-world connectivity dictates collective endothelial cell signaling

**DOI:** 10.1073/pnas.2118927119

**Published:** 2022-04-28

**Authors:** Matthew D. Lee, Charlotte Buckley, Xun Zhang, Lauri Louhivuori, Per Uhlén, Calum Wilson, John G. McCarron

**Affiliations:** ^a^Vascular Imaging Group, Strathclyde Institute of Pharmacy and Biomedical Sciences, University of Strathclyde, Glasgow G4 0RE, United Kingdom;; ^b^Department of Medical Biochemistry and Biophysics, Karolinska Institutet, SE-171 77 Stockholm, Sweden

**Keywords:** endothelium, network, signaling, small-world, calcium

## Abstract

Every blood vessel is lined by a single layer of highly specialized, yet adaptable and multifunctional endothelial cells. These cells, the endothelium, control vascular contractility, hemostasis, and inflammation and regulate the exchange of oxygen, nutrients, and waste products between circulating blood and tissue. To control each function, the endothelium processes endlessly arriving requests from multiple sources using separate clusters of cells specialized to detect specific stimuli. A well-developed but poorly understood communication system operates between cells to integrate multiple lines of information and coordinate endothelial responses. Here, the nature of the communication network has been addressed using single-cell Ca^2+^ imaging across thousands of endothelial cells in intact blood vessels. Cell activities were cross-correlated and compared to a stochastic model to determine network connections. Highly correlated Ca^2+^ activities occurred in scattered cell clusters, and network communication links between them exhibited unexpectedly short path lengths. The number of connections between cells (degree distribution) followed a power-law relationship revealing a scale-free network topology. The path length and degree distribution revealed an endothelial network with a “small-world” configuration. The small-world configuration confers particularly dynamic endothelial properties including high signal-propagation speed, stability, and a high degree of synchronizability. Local activation of small clusters of cells revealed that the short path lengths and rapid signal transmission were achieved by shortcuts via connecting extensions to nonlocal cells. These findings reveal that the endothelial network design is effective for local and global efficiency in the interaction of the cells and rapid and robust communication between endothelial cells in order to efficiently control cardiovascular activity.

## Significance

The endothelium is the single layer of cells lining all blood vessels and acts as a central control hub to regulate multiple cardiovascular functions in response to hundreds of physiological stimuli. The detection of various physiological stimuli is distributed in spatially separated sites across the endothelium. Distributed sensing is difficult to reconcile with the requirement for coordinated cell activity across large regions of the endothelium. Here, we show that the endothelium resolves the issue by using a network with scale-free and small-world properties. The organization confers a high signal-propagation speed and a high degree of synchronizability across the endothelium. The network organization also explains the robust nature of endothelial communication and its resistance to damage or failure.

The endothelium is a complex sensory and physiological control system that manages almost all cardiovascular functions. Endothelial control of cardiovascular function occurs by the release of numerous endothelial factors in response to countless circulating and local vasoactive agents that bring requests for changes in physiological status (1). Vasoactive agents may arrive simultaneously from hormones, neurotransmitters, pericytes, smooth muscle cells, various blood cells, viral or bacterial infection, proinflammatory cytokines, and mechanical stimuli (2–4). To manage the competing diversity of vasoactive agents, the endothelium reduces the complexity of the information presented by using clusters of cells that are specialized to detect specific activators (5–7). The clusters are separated spatially and appear as an irregular heterogeneous patchwork across small regions of the endothelium (8–11). Separate clusters tuned to particular agonists simplify detection by disentangling the complexity of multiple activators potentially acting on each cell. However, the segregation of detection sites introduces a significant problem of coordination of function across the endothelium. Coordination of function requires cells to respond together, an event unlikely to happen because of the spatial segregation of detection sites. Intercellular communication via gap junction and paracrine signaling facilitates information exchange (12–15); however, the precise organization of the network that critically determines how information is relayed and system behavior is controlled, is unknown.

Network organization controls system behavior by determining the overall intercellular signal propagation speed, the robustness of the system to failures and attack, and the degree of synchronizability in the system. Network (graph) theory is used to describe the organization and has gained much attention due to its ability to quantify social, technological, and biological systems. For example, analyses of networks have been used to predict the spread of disease through society (16, 17) and the complex flow of information through the World Wide Web (18). Examination of neuronal networks has revealed how information flow from a single, stimulated neuron can affect population-wide activity in vitro as well as in vivo (19). While complex networks are now recognized at all organizational levels in biological systems (20), no studies have examined the network structure employed by the endothelium in the control of cardiovascular function.

The ultimate goal of network analysis is to predict the spread of information across a system. Understanding how information is routed and communicated through complex systems requires mathematical methods that capture topological population connectivity including the extent of clustering (densely connected regions), average path length, the shortest number of connections between two points, and the presence of highly interconnected “hubs.” These features are critical in determining information dissemination, the tolerance of the system to failures and perturbations, and bottlenecks in information transfer. The arrangement also determines the control sites of the entire system that provide key targets for intervention when communication goes wrong (e.g., reduced cardiovascular function in disease).

**Fig. 1.**
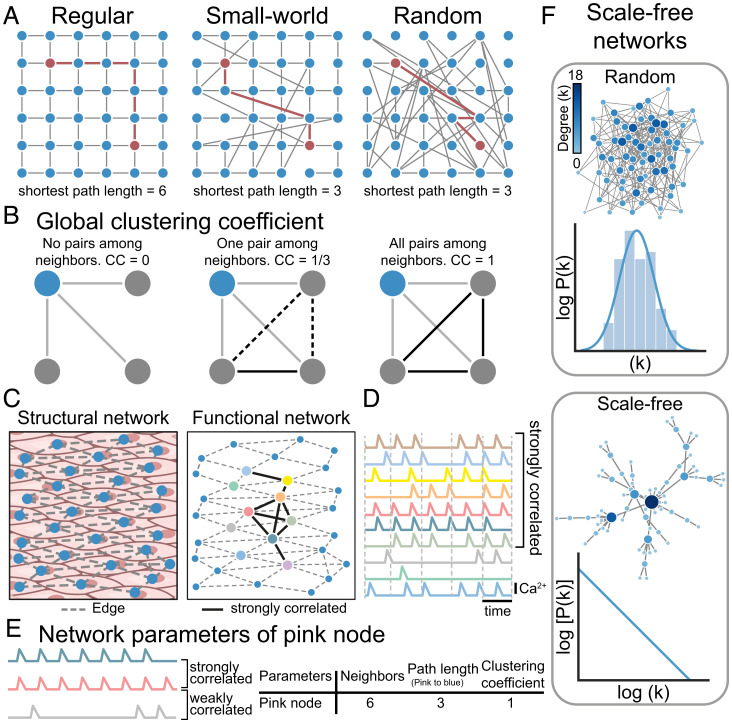
Parameters of the network structure. (*A*) Topologies of a regular, small-world, and random network with the shortest path length between the same two nodes for each network indicated (red line). (*B*) Clustering coefficient for the blue node is calculated for no connections between active neighbors, one connection between active neighbors, and all three connections between active neighbors. (*C*) Illustration of the structural (*Left*) and functional network (*Right*) of endothelial cells. The functional network operates on the structural network with links connecting the most correlated cells. (*D*) Pseudo Ca^2+^ responses from color-matched cells indicated in *C*. (*E*) The network parameters of the pink cell are indicated. The pink signal is shown with one strongly (blue) and one weakly (gray) correlated signal. (*F*) Schematic representation of random and scale-free networks, with degree distribution for each node indicated. Random networks follow a Poisson degree distribution with no hubs (nodes with high degree) present. Scale-free networks have a power-law degree distribution with hubs.

A network consists of nodes (individual endothelial cells in the case of the endothelium) and edges (the connection between the nodes; Fig. 1). Networks are classified on the basis of connections between nodes and range from fully ordered regular structures (lattice shape) to networks with connections of an entirely random organization (Fig. 1). In a regular network, all nodes have the same number of connections to nearest neighbors and give rise to well-organized behavior but at a significant temporal cost (21). Time scales operating in regular networks are not compatible with the fast signal processing required in many biological systems. Conversely, in a random network, a node is connected (randomly) to any other node within the system. In a random network, the number of connections in each node is distributed (Poisson) around some mean value. Random networks can give rise to rapid information transfer over distance but are unable to produce coherent synchronization of activity. Small-world networks share some functional features with random and regular network structures. Small-world networks are highly clustered, like regular lattices, but have the short path lengths that are characteristic of random graphs.

In another type of network, referred to as scale free, the connections between nodes are neither constant (as occurs in regular networks) nor randomly distributed (Fig. 1). Instead, there is an exponential relationship between the number of connections and its frequency of occurrence. Most nodes have very few connections, but a few are highly interconnected in the network. The uneven distribution of connections gives rise to nodes with unequal influence on behavior in the network. Nodes that are highly connected may act as hubs that can integrate and broadcast information widely. The presence of hubs is a major difference between random and scale-free networks.

To understand how activity in the endothelium is coordinated, we have used network analysis to determine the mechanism by which endothelial cells are able to rapidly respond to changes in the local environment but do so in a synchronized manner. Using Ca^2+^ signaling as a reporter of cell activity, we recorded the individual activity of ∼1,000 endothelial cells in intact resistance arteries and show that signaling in the endothelium operates on a small-world network. By combining features from regular and random networks, the endothelial small-world network gives rise to a fast system response with the facility for coordinated behavior (21). We also show that the distribution of the number of connections between active cells (the degree distribution) characterizes a scale-free network in which some endothelial cells are densely connected giving rise to cells that are candidates for hubs. The small-world, scale-free network topology enables the endothelium to detect and rapidly and separately respond to different activators. The organization also explains the stability and robustness of the endothelial system.

## Results

### Ca^2+^ Responses in Intact Endothelium.

**Fig. 2.**
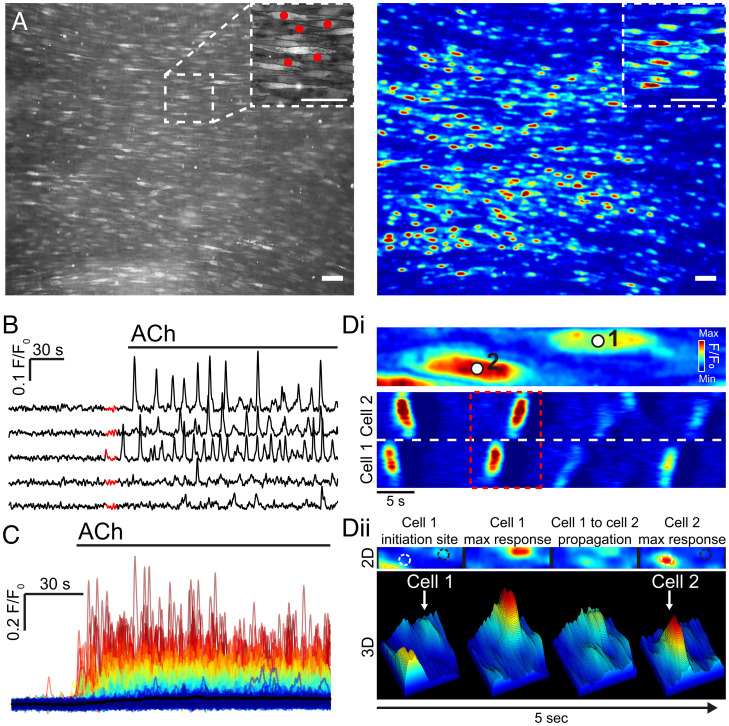
Ca^2+^ signaling in intact mesenteric endothelium. (*A*) Representative image showing ∼1,000 endothelial cells from an en face second order mesenteric artery (Left). ACh-induced (15 nM) Ca^2+^ activity from a 5-min recording (Right). (*B*) Baseline corrected Ca^2+^ signals (F/F_0_) obtained from the ROIs in (A, red dots). The highlighted section (red) on each individual Ca^2+^ signal indicates the software-determined baseline region. (*C*) Overlaid Ca^2+^ signals from all cells in A. The individual Ca^2+^ signals have been color-coded according to the amplitude of the initial rise in Ca^2+^; red indicates the highest amplitude and blue the lowest amplitude. (*D, i*) Zoomed region taken from A showing a 2D kymograph (linescan) of the Ca^2+^ signal intensity (color) plotted against time (x-axis) from the two cells indicated. (*D, ii*) A 2D and 3D surface plot showing signal propagation between the two cells highlighted in *B, i*. Scale bars, 50 µm.

The functionality of the endothelium requires a rapid and maintained flow of information among cells. Information passes from one cell to the next via connections in the endothelial network (22). The design of network connections is critical in determining communication efficiency, speed of information transfer, resistance to damage, and targets for manipulation of the system, but it is largely unknown. To understand the design of the network, we studied the structural and functional properties of endothelial communication in fields of ∼1,000 cells in intact mesenteric arteries using evoked Ca^2+^ responses. In the experiments, a region of interest (ROI) was generated for each of the ∼1,000 cells and the Ca^2+^ response (e.g., amplitude) from each cell was obtained (Fig. 2). Two agonists (acetylcholine [ACh] and histamine) were used at equivalent concentrations (25% effective concentration [EC_25_]) in these experiments. ACh and histamine were selected for study as representative endothelial activators. The response to muscarinic receptor activation is thought to underlie the response hypothermia (23) and to shear-stress activation (24), while histamine increases blood flow and endothelial permeability (25, 26).

First, to determine equivalent concentrations of each agonist, and the receptors involved in the responses, noncumulative concentration response curves for ACh and histamine were carried out. Each agonist produced widely distributed and heterogeneous responses across the field of endothelial cells that were concentration dependent (*SI Appendix*, Figs. 4 and 5 and Movies S1 and S2). The number of active cells and the amplitude of the Ca^2+^ response in each active cell increased with concentration (*SI Appendix*, Figs. 4 and 5). Ca^2+^ responses from activated cells propagated to neighbors creating a network of activated cells (Movies S1 and S2). The response to ACh was mediated by M3 receptors (inhibited by 4-DAMP; 100 nM) (*SI Appendix*, Fig. 6). The response to histamine was abolished by the H1 blocker triprolidine (100 µM) and unaffected by the H2 blocker cimetidine (100 µM) indicating histamine-evoked Ca^2+^ responses are mediated by H1 receptors (*SI Appendix*, Fig. 6).

The response to each agonist occurred by IP_3_-evoked Ca^2+^ release; there was little contribution from the ryanodine receptor (RyR) (*SI Appendix*, Fig. 7). The initial response to each agonist occurred by IP_3_-evoked Ca^2+^ release from internal stores, while the maintained response was due to Ca^2+^ influx across the plasma membrane (*SI Appendix*, Fig. 8).

### Network Structure.

**Fig. 3.**
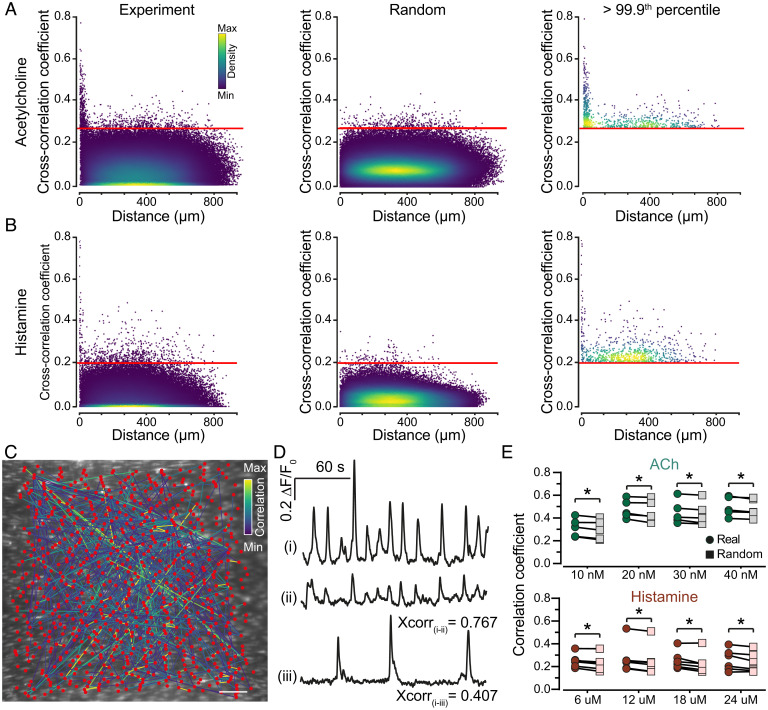
Network analysis of ACh- and histamine-evoked Ca^2+^ responses in the endothelium. Scatterplots of cross-correlation coefficients were plotted as a function of intercellular distance for experimental (Left) and random data (Middle) derived from ACh (*A*) and histamine-evoked (*B*) Ca^2+^ responses in the intact endothelium. Agonists were applied for 30 min, and the first 5 min of activation (in which an initial synchronized burst of activity occurred) was excluded from the analysis. The red line indicates the 99.9th percentile from the random data. (*C*) Network plot of cross-correlation coefficients greater than the 99.9th percentile for ACh. (*D*) Single-cell Ca^2+^ traces from two cells (*ii, iii*) that are strongly correlated to cell i (>99.9th percentile). (*E*) Cross-correlation coefficients as a function of agonist concentration for ACh (green) and histamine (red) from experimental and randomized data for data greater than 99.9th percentile. Data are representative of n = 5 independent experiments, from artery preparations, from different animals; *P < 0.05, paired Student t test. Scale bars, 50 µm.

Structurally, endothelial cells appear to form a regular lattice network, with each cell (node) connected (edges) to an average of six neighbors (*SI Appendix*, Fig. 2*E*). The communication network operates on this physical structure. To investigate communication network organization, we constructed graph theoretical representations of the underlying endothelial network structure (*SI Appendix*, Fig. 1). This approach permits formalisms of graph theory to analyze and understand the network system. The functional network was examined by activating endothelial cells with the EC_25_ concentration of ACh or histamine (Fig. 3).

Activation of 25% of cells with the EC_25_ was not always achieved precisely due to some variation in the sensitivity between preparations; therefore, analysis was restricted to the first 25% of cells that responded (when more than 25% of the cells responded; Fig. 2). Cross-correlation was used to determine the extent of connections between signals in cells throughout the population. Cross-correlation of paired cells was plotted as a function of distance between the two cells (Fig. 3). The mean of the 99.9th percentile of the randomized (scrambled) data was calculated for each agonist within each dataset (see *Methods*) and was used as a threshold value to determine connectivity (Fig. 3). Thus, two cells were defined as being functionally connected if their correlation coefficient exceeded the mean 99.9th percentile. A network plot of cell pairs with correlation coefficients above the cutoff showed clusters of highly correlated cells (Fig. 3*A*). No cell cluster formations were observed in a network plot of the scrambled data (Fig. 3*A*). Strongly correlated cells were closer to each other than weakly correlated cells (Fig. 3 *A* and *B*). This strong correlation between close cells did not occur in the scrambled data (Fig. 3 *A* and *B*). Using the data from cross-correlation analysis, features of the network structure were next determined.

### Network Features.

**Fig. 4.**
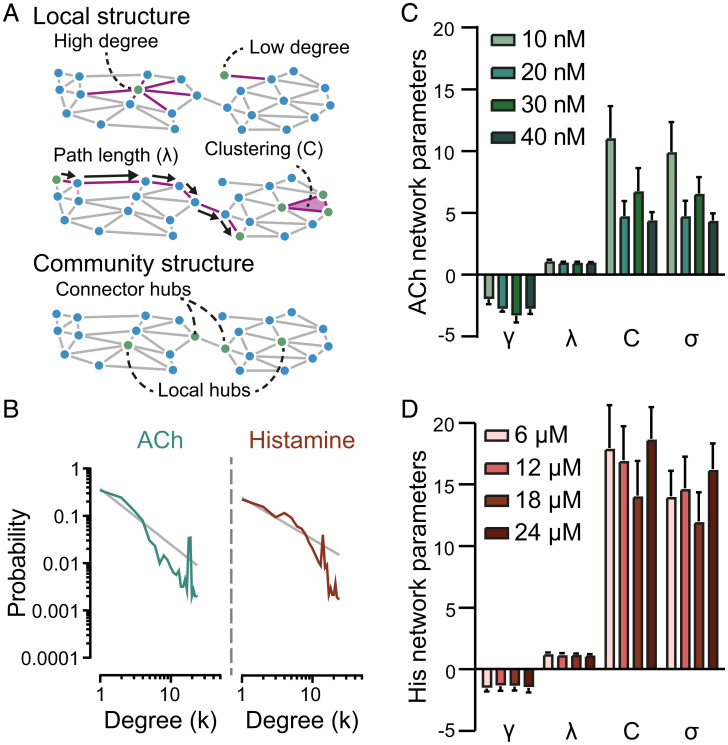
Network analysis summary. (*A*) Illustration showing network parameters. (*B*) Probability distribution for ACh (green) and histamine (red) plotted on a log-log scale with a linear regression fit in gray. (*C*, *D*) Summary data of network parameters. Slope (γ) is a connectivity measure that provides information on network structure. Mean path length (λ), cluster coefficient (C), and small-world coefficient (σ) are also shown at four agonist concentrations for ACh (*C*) and histamine (*D*). Data are representative of *n* = 5 independent experiments, from artery preparations, from different animals and means ± SEM values are shown in panels *C* and *D*.

A unique metric in network communication is a measure of the distance between nodes in information transfer (referred to as path length; Fig. 4). The average path length (λ, Eq. **1 **in *Methods*) is the shortest number of edges that must be traversed between pairs of nodes (for all node pairs) in the network. This value offers a measure of the network’s overall connectivity. In the endothelium, the average path length is strikingly small when compared to the network size (λ = 1.12 ± 0.1 for ACh and λ = 1.26 ± 0.1 for histamine; Eq. **1**; *n* = 5; Fig. 4). This value highlights the efficiency of endothelial communication.

**Fig. 5.**
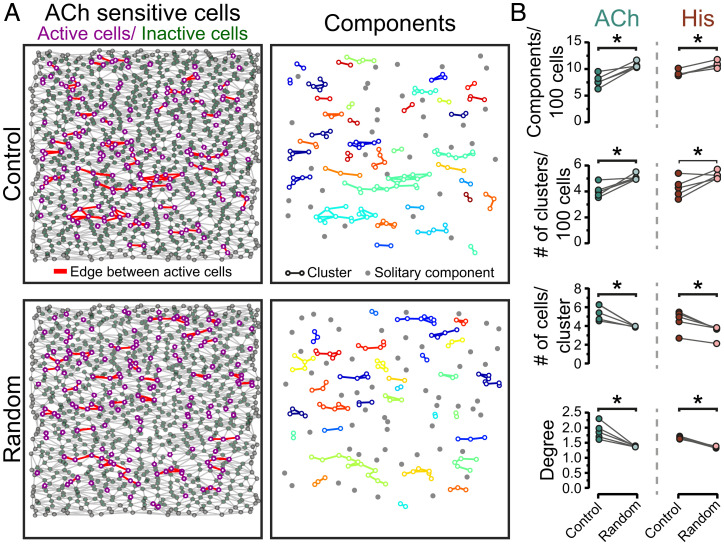
Clustering of ACh- and histamine-sensitive endothelial cells. The first 25% of responding cells to ACh (EC_25_) and their spatial relationships were calculated from neighbor analysis. (*A*) Reconstructed endothelial network (*Left*) showing locations of active cells (purple dots), inactive cells (green dots), and boundary cells (gray dots). Lines connect responding cells to their nearest responding neighbors (red) or nonresponding neighbors (gray). (*Right*) Shows the individual clusters (color) and isolated components (gray). Clusters are cells that respond with an active neighbor. (*Bottom*) A random model of the endothelial network shown in *A*. (*B*) Summary data illustrating the distribution of ACh- and histamine-sensitive cells across the endothelium compared to a random distribution. Degree is the average number of edges between an active cell and its active neighbors. Data are representative of *n* = 5 independent experiments, from artery preparations, from different animals; *P < 0.05, paired Student *t* test.

Another elementary network measure is the degree distribution. The number of edges (connections) a node has to other nodes in the network is referred to as its degree (Fig. 4), and the variability in degree between nodes is described by the degree distribution of the graph (fraction of nodes in the network with each degree, k). In the endothelial network, the degree distribution of active cells was higher than would be expected if responsive cells were distributed randomly throughout the endothelial network (ACh: 1.7 ± 0.01 measured vs. 1.3 ± 0.01 random, Fig. 5; histamine; 1.6 ± 0.1 measured vs. 1.3 ± 0.1 random, Fig. 5). However, the degree distribution is highly nonuniform and is not concentrated around a mean value. Instead, the endothelial network degree distribution was approximately linear on a log-log scale (slope (γ) = −1.99 ± 0.4 for ACh and γ = −1.56 ± 0.3 for histamine; Fig. 4). The linear relationship indicates a scale-free network structure, i.e., there is no typical node in the network that represents the degree for the other nodes. There are several implications of the scale-free organization. The characteristics of the network are independent of the size of the network, i.e., as the network grows, the underlying structure remains the same. The organization also is more stable and less vulnerable to disruption than other network organizations. In scale-free networks, cells with a high degree (connected cells; *k*) may function as hubs. In the endothelium, some cells were functionally connected to up 13 other cells (Fig. 4*B*). Increasing concentrations of the agonist had no effect on network parameters (Fig. 4*C* and *SI Appendix*, Fig. 9)

In some types of networks (e.g., small-world networks), there is a tendency of interconnected nodes to form clusters. To determine the extent of cell clustering in cells sensitive to ACh or histamine (EC_25_), the measured distribution of active nodes (cells) was compared to a randomized distribution of active cells in the same dataset (Fig. 5; see *Methods*). The extent of clustering of cells sensitive to each agonist (number of active cells in a cluster) was significantly higher than would be expected from a random distribution of the same cells (ACh: 5.2 ± 0.09 measured vs. 3.8 ± 0.06 random, Fig. 5 *A* and *B*; histamine: 4.7 ± 0.36 measured vs. 3.8 ± 0.12 random, *n* = 5; Fig. 5*B*). The number of clusters per 100 cells (i.e., cells that responded with at least one responding neighbor) was significantly higher in the random model for ACh and histamine when compared to the measured data (ACh: 3.5 ± 0.09 measured vs. 4.1 ± 0.07 random, Fig. 5; histamine: 3.6 ± 0.12 measured vs. 4.0 ± 0.10 random, Fig. 5). These results suggest that responding cells are grouped together into sensory clusters, i.e., more cells in a cluster, resulting in fewer clusters, when compared to a random arrangement.

In a network, the clustering coefficient (the number of triangles within a set of nodes divided by the total number of possible connected triplets for that dataset) can be used to determine the extent to which nodes tend to cluster. The global clustering coefficient (C; Eq. **2** in *Methods*) corresponds to the measured clustering coefficient divided by the clustering coefficient derived from a scrambled version of the same data (see *Methods*). In the endothelium, the high global clustering coefficient for ACh (11.1 ± 2.6) and histamine (17.8 ± 3.5) confirms the clustered organization of signal detection systems in the endothelium.

**Fig. 6.**
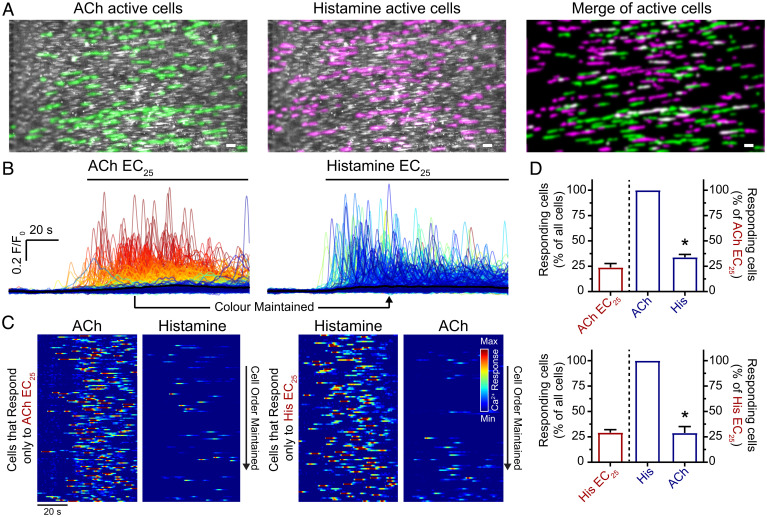
Ca^2+^ activity evoked by ACh and histamine occur in spatially discrete cells. (*A*) Composite endothelial Ca^2+^ image (second-order mesenteric artery, ∼1,000 cells) with activity evoked by the EC_25_ of ACh (green) and histamine (magenta) overlaid in the same field of endothelium. (*B*) Cellular Ca^2+^ signals from respective dataset shown in *A*. Ca^2+^ signals have been colored according to the magnitude of the response to ACh (red [highest] to blue [lowest]) and the color (identity) of each trace has been maintained across the histamine dataset (i.e., if a cells’ trace is colored red for ACh then it is also colored red for histamine). (*C*) Heatmap representation of Ca^2+^ signals, of the active signals, shown in *B*. Each line represents the Ca^2+^ response from a separate endothelial cell. (*D*) Summary data showing the percentage of the total population of cells activated at the EC_25_ (red) and the percentage of these same cells (active; red bar) that are activated by the other agonist (blue). Data are representative of *n* = 5 independent experiments, from artery preparations, from different animals; *P < 0.05 compared to EC_25_ response, paired Student *t* test. Scale bars, 50 µm.

Furthermore, largely separate populations of cells were activated by each agonist. ACh (EC_25_) evoked a response in 23.8 ± 3.9% of all cells. Of these ACh-activated cells, only a small portion responded to histamine (33.9 ± 2.8%) (Fig. 6). Of the cells responding to the EC_25_ of histamine (29.2 ± 3.0%), a small subpopulation responded to ACh (28.8 ± 6.3%) (Fig. 6). These results show that there are separate populations activated by each agonist. Collectively, these results suggest that agonist-sensitive endothelial cells are positioned in spatially distinct clusters throughout the endothelium.

The spatial pattern of cells activated by each agonist was highly reproducible and was independent of the sequence of agonist application (Movie S3 and *SI Appendix*, Fig. 10). Together, these observations indicate that the endothelium uses a network of spatially distinct clusters of cells that are primed to detect a specific agonist.

### Network Characteristics.

Our results show that the endothelium employs high clustering and short path lengths in signal detection and communication. These are features that characterize small-world networks. In small-world networks, most nodes (cells) are not neighbors, but the neighbors of any given node can be reached from every other node by a small number of steps. To test if the endothelium is a small-world network, the small-world coefficient (σ; Eq. **3** in *Methods*) was calculated. The small-world coefficient compares the global clustering coefficient (*C*; Eq. **2**) and average path length (λ) of a given network to an equivalent random network with same degree on average. A network is considered small-world if σ (Eq. **3**) is greater than 1. The results show that ACh- and histamine-sensitive cells, although spatially distinct, each operate on a small-world network to detect and communicate across the endothelium (σ =9.9 ± 2.4 for ACh and σ = 13.9 ± 2.1 for histamine; Fig. 4).

To provide an additional test of cell-cell communication, we locally activated preselected populations of cells and examined signal propagation to neighboring quiescent cells (*SI Appendix*, Fig. 11). To do this, the M3 receptor inhibitor 4-DAMP (100 nM) was applied to ∼50% of the artery (using local application via a puffer pipette against a flow of physiological saline solution [PSS] to limit 4-DAMP diffusion) and the position of 4-DAMP was determined using a fluorophore (sulforhodamine B; 2 µM) to identify receptor-blocked cells (*SI Appendix*, Fig. 11). After the receptor block, the artery was activated by ACh. A substantial propagation of signals (up to several hundred microns) occurred into the region that was blocked by 4-DAMP. Furthermore, the propagated signals were subsequently blocked when the IP_3_ receptor inhibitor caffeine (20 mM) was included in the puffer pipette. These results demonstrate that cell-cell communication occurs as cell activity propagated into cells that were blocked from direct agonist activation. The global application of caffeine to the entire preparation subsequently inhibited all ACh-evoked Ca^2+^ signals (*SI Appendix*, Fig. 11*C*).

**Fig. 7.**
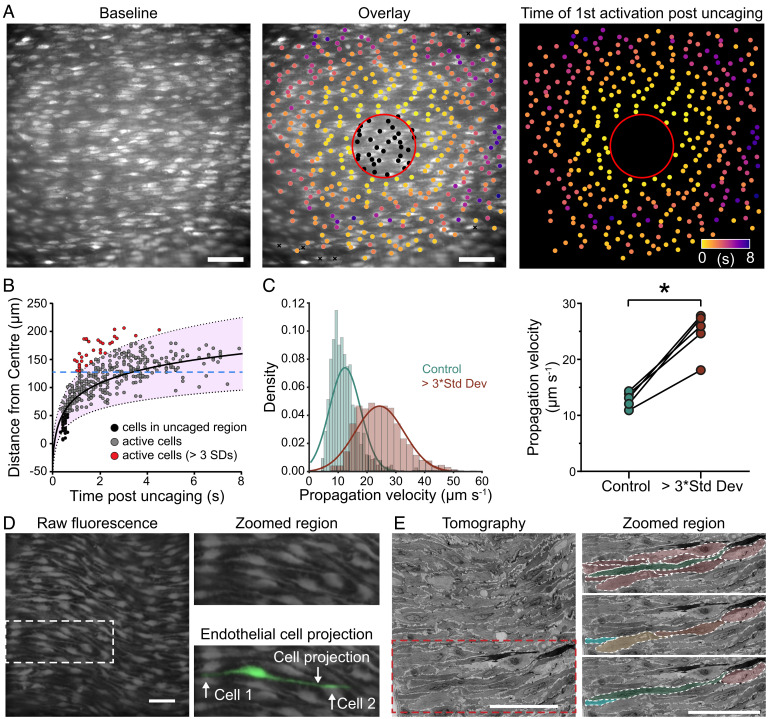
Endothelial cell projections permit rapid signal communication. (*A*) Representative composite image of Ca^2+^ activity in an en face second-order mesenteric artery endothelium to IP_3_ uncaging (5 μM, 30-min incubation). The heatplot shows the time of activation post uncaging. (*B*) Scatterplot of distance from the uncaging site plotted against time postuncaging. Cells that respond quickly but at significant distance from the uncaging region (three times the SD of the mean distance) are shown in red. (*C*) A distance threshold was set as the minimum distance required for an active cell to exceed three times the SD, for each dataset (shown as a blue line in *B*). Histogram (*Left*) and summary data (*Right*) of propagation velocity of cells that exceeded this threshold distance and do (red)/do not (green) exceed three times the SD. (*D*) Intercellular propagation may be facilitated by extended endothelial cell projections. (*E*) Electron tomographs showing projections that extend significant lengths. Data are representative of *n* = 5 independent experiments, from artery preparations, from different animals; *P < 0.05 compared to EC_25_ response, paired Student *t* test. Scale bars, 50 µm.

The small-world network structure facilitates rapid information transfer. To determine if rapid communication does indeed occur, we locally activated populations of cells using focal photorelease of caged IP_3_ and then examined the outward propagation of the Ca^2+^ wave (Fig. 7 and Movie S4). After localized photorelease of IP_3_, a rise in Ca^2+^ occurred in the population of activated cells that then propagated and expanded outward from the photolysis site in an approximately circular pattern. The outward propagation reflects cell-cell (intercellular) communication to cells not activated by the initiating stimulus (photoreleased IP_3_). While the intercellular wave propagated at an approximately constant speed, several cells activated at significant distances ahead of the wave front (Fig. 7 *A* and *B*) to generate a rapid short-circuit form of communication (Fig. 7*C*).

The question arises as to how the rapid short-circuit communication occurs. While each cell appears to have an average of 6 neighbors, close examination of the structural organization of cells revealed that some endothelial cells have exceptionally long processes (Fig. 7*D*) that will provide a mechanism for long distance transmission. Indeed, electron tomographs show the extended nature of the connections between some cells to provide distant connections (Fig. 7*E*).

## Discussion

Our results reveal that cells in the endothelium are structured for signaling in a functionally more efficient way than currently appreciated. Our findings show that intercellular signaling in the endothelium uses a highly ordered network that follows a scale-free topology with a small-world configuration. Scale-free and small-world networks confer local and global efficiency in the interaction of cells and are characterized by high signal-propagation speed and a high degree of synchronizability (21, 27) that permits function to be coordinated. This network arrangement also accounts for the robust and stable endothelial communication system that is resistant to attack and damage.

Information flow is critical for the endothelium to process the endless instructions that target various functions required to regulate cardiovascular activity. In carrying out this role, the endothelium is often treated as being a homogenous population of cells in which each cell responds identically to various stimuli. However, with developing imaging technologies, it has become clear that the endothelium is highly heterogeneous and uses numerous, separate clusters of cells that are specialized to detect specific signals (5, 8, 28–31). This heterogeneous arrangement reduces the complexity in the information presented to the endothelium by using clusters of cells that are specialized to detect specific stimuli, rather than each cell detecting each of the multiple stimuli present (28, 29). In addition, cells that detect specific stimuli, within a population of endothelial cells, have varying ranges in activation thresholds and saturation levels. This permits the detection of a stimulus across various intensity levels. Therefore, as stimulus intensity increases, cells of progressively higher thresholds will be recruited extending the monitoring sensitivity range of the entire population (5, 32).

However, while using specialized clusters of cells enables numerous inputs to be processed in parallel, the clusters are separated spatially. As a result, sensing of physiological stimuli is distributed in what initially appears as a disconnected patchwork across the endothelium (8–11). This spatially separated detection system may appear at odds with the requirement for coordinated endothelial cell activity to control cardiovascular function. The endothelium resolves this problem by using a network configured to have short average path lengths that results in rapid information spread. The short average path length in the endothelial network may arise from “short-cut” connections between cells. Evidence for the short-cut was found in local photoactivation to generate Ca^2+^ signals in small groups of cells. Ca^2+^ signals moved outward from the activation site at an approximately constant speed but, at times, appeared to skip ahead of the wave front and arrive early at distant sites to provide a rapid signal transmission. The route for the short-cut may involve long connective processes that occur among some endothelial cells.

The endothelium thus has a high global clustering of sensory cells and low average path lengths; these features characterize a small-world network. In the present study, the global clustering coefficient and path length of the endothelial network were compared to the equivalent parameter in a random network. We show muscarinic- and histaminergic-evoked responses each operate on a small-world network (σ = 9.9 for ACh and σ = 13.9 for histamine). The endothelial small-world connectivity supports efficient information segregation and integration with low wiring costs, efficient parallel processing, and facility for rapid adaptive reconfiguration in damage or attack (33, 34). Together, these features of a small-world network are well suited for the complex information handling required in the endothelium.

Our network analysis also revealed the number of connections each cell possesses (degree distribution) scaled to a power-law (k^−γ^) distribution. The power-law degree distribution revealed a continuity of connectivity. Most nodes (cells) have only a few edges (connections) between active nodes, however, a few nodes have a disproportionately large number of connections. Nodes with a large number of edges are often referred to as hubs and may act to link multiple low degree nodes together. These hubs are sites of information gathering and distribution. Our analyses revealed some cells were functionally highly connected to up to 13 other cells, even though cells on low resolution imaging appear structurally connected to only 6 neighboring cells. The long tails projecting among cells may provide the extended connections.

Graph theory predicts that small-world network designs are effective for biological systems, as they enable efficient information transfer and robustness against damage or failure (35, 36). The robustness to damage arises from the scale-free network topology and its hub-based small-world backbone. In this arrangement, deletion of random nodes rarely alters communication across the system as the mean shortest path length is not significantly increased. Nor does deletion of a random node decrease the clustering coefficient. Indeed scale-free networks have been shown to maintain their structural integrity even after the deletion of as many as 80% of their nodes (35, 37). This stability arises because the vast majority of nodes in scale-free networks have only one or two links and play a marginal role in maintaining the functional integrity of the network. Most random failures will occur on minimally connected nodes and will as a result not significantly disrupt the network’s information flow. An intuitive example of how small-world, scale-free networks function is found in how airline flights (edges) connect airports (nodes) across the world. A random disruption to one airport would not usually disturb the global flow of travelers. However, a shutdown of a major hub (e.g., Heathrow Airport) would severely disrupt the network. Hence, small-world, scale-free networks are robust against the random failure of nodes but are vulnerable to an impairment of hubs. Small-world, scale-free network architectures are found in large metabolic networks (38, 39) and neural networks (40–42).

While the network structure is resilient against damage, change nonetheless occurs during disease and significantly impacts the function of the system (e.g., increased path length or altered clustering). For example, in obesity, the deficient endothelial control of vascular tone occurs due to impaired population-level Ca^2+^ signaling (43). Obesity alters the endothelial network so that endothelial cells are reorganized into fewer but larger clusters, resulting in an increase in communication path length between clusters (43). These changes in endothelial cell network dynamics impair the collective endothelial response. Such perturbation patterns are of crucial importance in understanding network communication and coordination during a disease state in which the behavior of individual receptors appears unaltered but the overall tissue response is changed. An appreciation of the significance of the changes to network parameters may give the basis for understanding the functional differential expression patterns and observed changes in disease states (44) to permit the development of therapeutic targets.

Hubs and distributed activity in the network are key to the stability of the system. However, control is required so that activity in the system can be changed. Network controllability is the ability to guide a system’s behavior and arises by the management of inputs. While hubs are key to stability, they may play only a minor role in a system’s controllability (45, 46). Rather, control may be determined by a relatively few number of “driver nodes” (45), in a process that is not fully understood. These driver nodes are capable of steering a system to a desired state. The number of driver nodes in a system is dependent on edge dynamics and average degree distribution. A network with relatively few (or no) variations in degree distribution (i.e., regular or random networks) will require only a small number of driver nodes to control the system. However, networks with a large difference between node degrees require many more driver nodes in order to control the system. Indeed, small changes in the degree distribution increase the density of driver nodes by orders of magnitude (45). Scale-free networks, like the endothelium, are highly nonregular and require many more driver nodes to control system dynamics when compared to a regular network structure. A key step in therapeutic development is identifying these driver nodes that offer control of the system. Specific targeting of these driver nodes, by therapeutic intervention, may allow the system to be steered toward a predisease state. Network analysis offers the potential for the identification of these driver nodes.

Collectively, our results indicate that cells in the endothelium do not act individually or as a uniform collective. Instead, they work together in a cooperative way to provide small specific elements of the overall information available. This information is disseminated across the endothelium by use of a small-world, scale-free network. Understanding the relationships among cell interactions is a significant challenge. New analytical approaches are required for identifying key cells (or clusters) that govern the endothelial behavior critical for normal function and that drive the development of medical conditions.

## Methods

### Animals and Artery Dissection.

All animal care and experimental procedures were carried out with the approval of the University of Strathclyde Animal Welfare and Ethical Review Board (Schedule 1 procedure; Animals [Scientific Procedures] Act 1986, UK), under UK Home Office regulations. All experiments used freshly isolated mesenteric arteries obtained from male Sprague-Dawley rats (10 to 12 wk old; 250 to 350 g) euthanized by CO_2_ overdose. The mesenteric bed was removed and placed in physiological saline solution (PSS; composition below).

Intact second- or third-order mesenteric arteries (<250-μm outer diameter) were isolated from the mesenteric bed, cleaned of connective tissue and fat, cut open using microscissors, and pinned to a custom flow chamber with a Sylgard bottom, containing PSS.

### Ca^2+^ Imaging.

Arteries were incubated in a loading solution containing a fluorescent Ca^2+^ indicator, Cal-520 acetoxymethyl ester (Cal-520/AM; 5 µM), 0.02% Pluronic F-127, and 0.35% dimethyl sulfoxide in PSS for 30 min (37 °C). All Ca^2+^ measurements were carried out in PSS. Cal-520 was excited with 488-nm wide‐field epifluorescence illumination provided by a light-emitting diode illumination system (PE-300Ultra, CoolLED) and imaged using an EMCCD camera (13-µm pixel size iXon Life 888; Andor), through a 16× (water immersion; numerical aperture of 0.8; Nikon CFI75) objective lens or 40× (water immersion; numerical aperture of 0.8; Nikon CFI Apo) objective lens. Fluorescence emission was recorded at 10 Hz. Fluorescence illumination was controlled, and images (16-bit depth) were captured by µManager.

Ca^2+^ imaging recordings were analyzed using custom FIJI macros and custom analysis software written in the Python 2.7 programming language (5, 24). ROIs were automatically generated for each endothelial cell so that a direct comparison of the Ca^2+^ activity in each cell between various pharmacological applications could be made (24, 47).

### Localized IP_3_ Uncaging.

In experiments in which endothelial Ca^2+^ responses were evoked by photolysis of caged IP_3_, the endothelium was loaded with Cal-520/AM and a membrane-permeant Ins(1, 4, 5)P3-caged IP3 (cIP_3_; 5 µM for 30 min at 37 °C) (48, 49). Photolysis of cIP_3_ was achieved using a Rapp Optoelectronics flash lamp (00-325-JML-C2) at 300 V, which produced light of ∼1-ms duration. The flashlamp output was passed through a 395-nm short pass filter into a 1,250-µm-diameter light guide and then through a 40× 1.4NA objective lens. The light guide was coupled to the epiilluminator of the TE300 microscope, and the output was focused on the endothelium using broadband light. For each imaging session, broadband light was used to identify the position of the uncaging region (∼70-µm diameter) and to determine which endothelial cells were directly activated by the spot photolysis system.

### Local Agonist Activation.

In an additional approach to study cell-cell communication in the endothelium, the muscarinic blocker 4-DAMP (100 nM) was used to selectively block direct agonist-evoked responses in a subpopulation of endothelial cells. To do this, 4-DAMP was applied by pressure ejection from a puffer pipette, against a stream of PSS flow to a preselected endothelial region. The stream of PSS flow was used to restrict the diffusion of the blocker. The fluorescent dye sulforhodamine B (2 µM) was included in the puffer pipette with 4-DAMP to identify the cells blocked by the antagonist.

ACh (100 nM) was applied to the artery, and the Ca^2+^ response evoked in cells that were blocked with 4-DAMP was analyzed as described above. In the same artery and field of view, caffeine (20 mM) and the fluorescent dye sulforhodamine B (2 µM) were applied to the endothelium using a puffer pipette. Again, ACh was applied to the artery, and the Ca^2+^ responses evoked in the endothelium were analyzed. The presence of the fluorescent dye allowed mapping of the regions blocked by caffeine to the regions blocked by 4-DAMP.

### Cell Clustering and Neighbor Analysis.

To quantitatively study endothelial cell connectivity and network structure (Fig. 3), we generated empirical representations of the endothelial network using graphs in Python 2.7. To generate accurate ROIs for each cell in the field of view, cell markers along the length of each individual endothelial cell were created in FIJI. The dilate no merge plugin in FIJI expanded each of these lines simultaneously until they touched another (dilating) line or reached the boundary of the field of view (50). Cells at the boundary of the field of view have unknown connectivity and were therefore excluded from analysis. To determine the boundary of the field, a set of coordinates were created that contained the center point of each line (through the cell), and the corresponding concave hull (alpha shape) was calculated (51) (*SI Appendix*, Fig. 1). ROIs with a vertex that extended beyond this envelope were considered a boundary cell.

The minimum distance between each ROI (cell) and every other ROI was measured, and cell pairs with a separation of zero were classified as neighbors. The structural network was represented as graphs using the NetworkX library in Python 2.7 (52). Graphs allowed Ca^2+^ responses from individual cells to be compared with the Ca^2+^ response evoked in all other endothelial cells.

### Network Organization of Cells Responding to Each Agonist.

To determine the extent of clustering of agonist-sensitive endothelial cells, graphs of the distribution of cells responding to each agonist were created (*SI Appendix*, Fig. 2). For this analysis. the EC_25_ was used for each agonist i.e., the 25% most sensitive cells were identified. This value was selected as a number that generated activation of a significant population of cells but was low enough to avoid saturated activation of cells and wide-scale communicated recruitment. The following parameters were determined to assess the extent of cell clustering: 1) number of components (components are cells that respond in isolation or together with a neighbor), 2) number of clusters (clusters are cells that respond with an active neighbor), 3) the number of cells per cluster, and (4) the degree distribution (the average number of edges [connections] between an active cell and its active neighbors).

To determine the extent of statistical confidence and whether or not endothelial clustering was greater (or less) than expected from randomly positioned cells, a permutation analysis was used. This analysis compared the measured distribution to a completely randomized distribution of the same population. To do this, the Ca^2+^ response from each active cell was randomly assigned to any other cell (excluding boundary cells) to generate a new graph of active cells. This process was repeated 1,000 times for each dataset, and the mean number of components, number of clusters, number of cells per cluster, and degree distribution was calculated each time.

### Cross-Correlation Analysis.

Cross-correlation was used to determine whether two cells were functionally connected (*SI Appendix*, Fig. 3) (40, 53). Cross-correlation is a measure of the relationship between two signals. Here, discrete derivative Ca^2+^ traces were each converted to binary signals by assigning all time points in the differentiated signals above the threshold (fivefold SD of baseline noise) to 1 and all other points to zero (*SI Appendix*, Fig. 3). The cross-correlation coefficient for any pair of active cells could vary depending on the length or portion of the recording; therefore, a sliding window correlation was used. In the sliding window correlation, the binary signals were split into two portions of equal length (60 s) after the onset of stimulated Ca^2+^ activity (*SI Appendix*, Fig. 3 *B*–*D*). Two signals may be highly correlated even when there is a difference in the time of activation. The calculation of the correlation as a function of lag enables the determination of the maximum correlation irrespective of lag. Here, we used a lag of 2.5 s (*SI Appendix*, Fig. 3*B*). In brief, the maximum cross-correlation of respective portions of each signal were calculated by measuring the correlation between portions as one was shifted in increments of 0.1 s from −2.5 s to +2.5 s. For each neighboring cell pair, the mean of the cross-correlation coefficients was used as a measure of coupling between cells. A correlation coefficient equal to 0 indicates no linear relation between the waves, whereas a coefficient equal to 1 or −1 demonstrates a perfect linear relation. Calculating the correlation as a function of lag enables a determination of the maximum correlation despite lag (*SI Appendix*, Fig. 3*B*). The correlation function is amplitude independent and only considers a binary signal (0 or 1) (*SI Appendix*, Fig. 3*A*). The process was repeated for each pair of active cells.

To test cross-correlation statistical significance, a scrambled dataset was created for each cell pair. This process was undertaken to break any temporal link (communication) between the signals occurring in one cell and another (*SI Appendix*, Fig. 3*C*). To create a scrambled dataset, the timing of occurrence of the signals from one cell of each cell pair was disrupted by selecting a portion of the Ca^2+^ signals at a random time point and moving those signals to the start of the original signal (*SI Appendix*, Fig. 3*C*). The cross correlation was again calculated for each cell pair after signal translation. This process was repeated 100 times for each dataset. A significant advantage of this method is that the total activity in the original data set and the scrambled data set are conserved in each iteration and the underlying total cell numbers and cell arrangements are constant. This process was used to determine a cutoff that filters out insignificant correlations. A pair of cells was defined as being functionally connected if the cross-correlation coefficient was greater than the 99.9th percentile of the scrambled data for each dataset.

#### Graph Theory.

Graph theory was used to characterize the topology of a network (19). In graph theory, several network parameters are calculated for each network to determine the network’s type (54) (Fig. 3). Several network properties were calculating to determine the network type (Newman, 2010; *Networks: An Introduction*. Oxford: Oxford University Press.). These properties include the mean shortest path length (λ, Eq. **1**) and mean global clustering coefficient (C, Eq. **2**). The C corresponds to the number of triangles within a set of nodes divided by the total number of possible connected triplets for that dataset. The mean shortest path length is the minimum number of edges (L) that must be traversed to travel from one node to another (Fig. 3 *A* and *C*). The values of C and L were compared to the corresponding C_R_ and L_R_ values for a randomized version of the network. To generate a random version of the network, nodes were randomly selected as active (limited to the same number as in the observed data), and cross-correlation analyses (as described above) were performed. This was repeated 100 times, and the grand mean of both the mean cross-correlation coefficients (C_R_) and mean shortest path length (L_R_) for each random network was measured. A network is considered to have small-world (σ; Eq. **3**) properties if:

[1] $$\lambda =\frac{L}{L_{R}}\approx 1$$

[2] $$C=\frac{C}{C_{R}}>1$$

[3] $$\sigma =\frac{\frac{C}{C_{R}}}{\frac{L}{L_{R}}}>1$$

Classical models of networks are either regular or random (Fig. 3*A*). In a regular network, each node is connected to k other nodes (Fig. 3). In a random network, nodes are connected with links stochastically, resulting in a Poisson-shaped degree distribution (Fig. 3*F*). Small-world networks combine features of both regular and random networks and have high clustering, as occurs in regular networks, but with short internodal distances characteristic of random networks (Fig. 3).

### Agonist-Evoked Endothelial Ca^2+^ Responses.

Full noncumulative concentration responses were obtained for each agonist, in the same field of endothelium in a single mesenteric artery preparation (*SI Appendix*, Figs. 4 and 5). After each agonist concentration application, arteries were washed with PSS for 5 min and allowed to re-equilibrate. A stable baseline was attained before the subsequent addition of an agonist.

Agonist and antagonists were used to determine the mechanisms involved in muscarinic- and histaminergic-evoked endothelial Ca^2+^ responses. Agonists were applied to the artery before and after incubation with the inhibitor/modulator. Incubation times are indicated in the text. Each inhibitor/modulator remained present throughout all subsequent additions of agonist. Ionomycin (1 µM) was applied at the end of each experiment to confirm endothelial cell viability.

The contribution of Ca^2+^ influx in muscarinic and histaminergic endothelial Ca^2+^ signaling was determined by removing extracellular Ca^2+^. Arteries were perfused in Ca^2+^-free PSS (containing 1 mM ethylene glycol-bis(β-aminoethyl ether)-N,N,N′,N′-tetraacetic acid [EGTA]), and after 60 s, the agonist was applied to the endothelium. Arteries were perfused with normal PSS, between each application of Ca^2+^-free PSS and agonist, to allow the internal store to refill before subsequent activations.

### Electron Microscopy.

Samples were pinned out on Sylgard blocks, cut open longitudinally, and pinned out in an en face preparation. Samples were then transferred to 4% paraformaldehyde/2.5% glutaraldehyde in 0.1M phosphate buffer (72 mM Na_2_ HPO_4_ 0.2 H_2_O, 28 mM NaH_2_PO_4_ 0.2 H_2_O in distilled water [pH 7.2]) for 4 h at room temperature and then stored in 1:10 solution at 4 °C prior to processing. Processing was carried out in the Pelco Biowave Pro+. Samples were washed in 100 mM cacodylate buffer followed by secondary fixation and heavy metal staining with 1% OsO_4_ and 0.5% K_3_Fe(CN)_6_. Samples were then washed with dH_2_O followed by dehydration with ethanol (50, 70, 95%) and then acetone prior to gradual infiltration of Spurr’s resin (TAAB) (10, 30, 50, 70, 90% in acetone). After infiltration was completed, samples were cut from Sylgard blocks and embedded. The resin was polymerized in a 60 °C oven for 24 h. Next, 250-nm sections were cut using a Leica UC6 ultramicrotome and Diatome diamond knife, and sections were collected on 100 square mesh grids and counterstained with Uranyless (TAAB) and Ultrostain 2 (Leica) using a Leica AC20. Tomograms were acquired on a Jeol 1400+ with an AMT UltraVUE camera.

### Solutions and Drugs.

Cal-520/AM was obtained from Abcam. Pluronic F-127 was obtained from Invitrogen. U73122, and U73343 were obtained from Tocris Bioscience. All other drugs and chemicals were obtained from Sigma-Aldrich. PSS consisted of the following: 145 mM NaCl, 4.7 mM KCl, 2.0 mM 3-(N-morpholino)propanesulfonic acid (MOPS), 1.2 mM NaH_2_PO_4_, 5.0 mM glucose, 0.02 mM ethylenediaminetetraacetic acid (EDTA), 1.17 mM MgCl_2_, and 2.0 mM CaCl_2_, adjusted to pH 7.4 with NaOH. Ca^2+^-free PSS consisted of the following: 145 mM NaCl, 4.7 mM KCl, 2.0 mM MOPS, 1.2 mM NaH_2_PO_4_, 5.0 mM glucose, 0.02 mM EDTA, 1.0 mM EGTA, and 2.34 mM MgCl_2_, adjusted to pH 7.4 with NaOH. Ryanodine, 2-aminoethoxydiphenyl borate (2-APB), and cyclopiazonic acid (CPA), were dissolved in dimethyl sulfoxide. All solutions were prepared each day.

### Statistics.

Summarized data are presented as means ± SEM values; n refers to the number of animals. Data were compared as indicated using a two-tailed Student’s *t* test (paired data) or a two-tailed one-way ANOVA with Tukey’s multiple-comparisons test, as appropriate. Sigmoidal curves were fitted to concentration response data, and the minima of the sigmoidal curves were constrained to zero. All statistical analyses were performed using GraphPad Prism, version 6.0 (GraphPad Software). A *P* value of <0.05 was accepted as statistically significant.

## Supplementary Material

Supplementary File

Supplementary File

Supplementary File

Supplementary File

Supplementary File

## Data Availability

All study data are included in the article and/or supporting information.
