# Supplementary material for: Small-world connectivity dictates collective endothelial cell signaling
Source: Proc Natl Acad Sci U S A. 2022 Apr 28;119(18):e2118927119. doi: 10.1073/pnas.2118927119 (PMC9170162; doi:10.1073/pnas.2118927119)
Supplement: Supplementary File [file pnas.2118927119.sapp.pdf]

SUPPLEMENTARY INFORMATION

SUPPLEMENTARY FIGURES

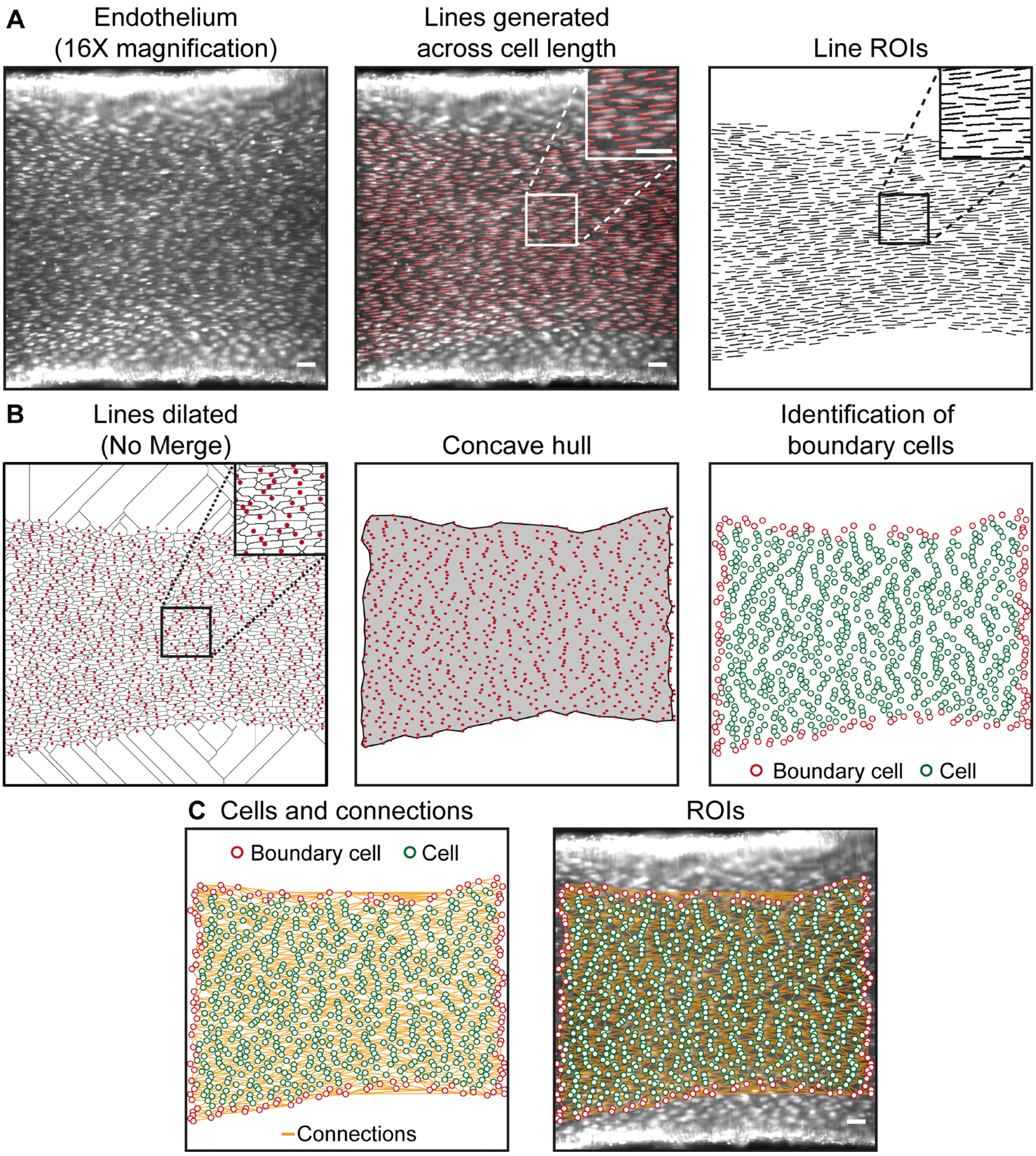

Supplementary Figure 1 – Reconstructing endothelial network connectivity from Ca^{2+} imaging recordings.

(A) Representative Ca^{2+} image (left), showing ~1000 endothelial cells, of an *en face* second-order mesenteric artery. Line ROIs drawn along the length of each individual cell (middle and right) used to create cell outlines. (B) Cellular ROIs (left) generated using the “dilate no merge” plugin in FIJI. (Middle panel) A concave hull was applied to determine the boundary of the preparation, encompassing all cells. The concave hull was used to identify cells at the boundary of the network (or field-of-view; right). (C) Reconstructed endothelial network (left) overlaid on Ca^{2+} image (right). Scale bars, 50 µm.

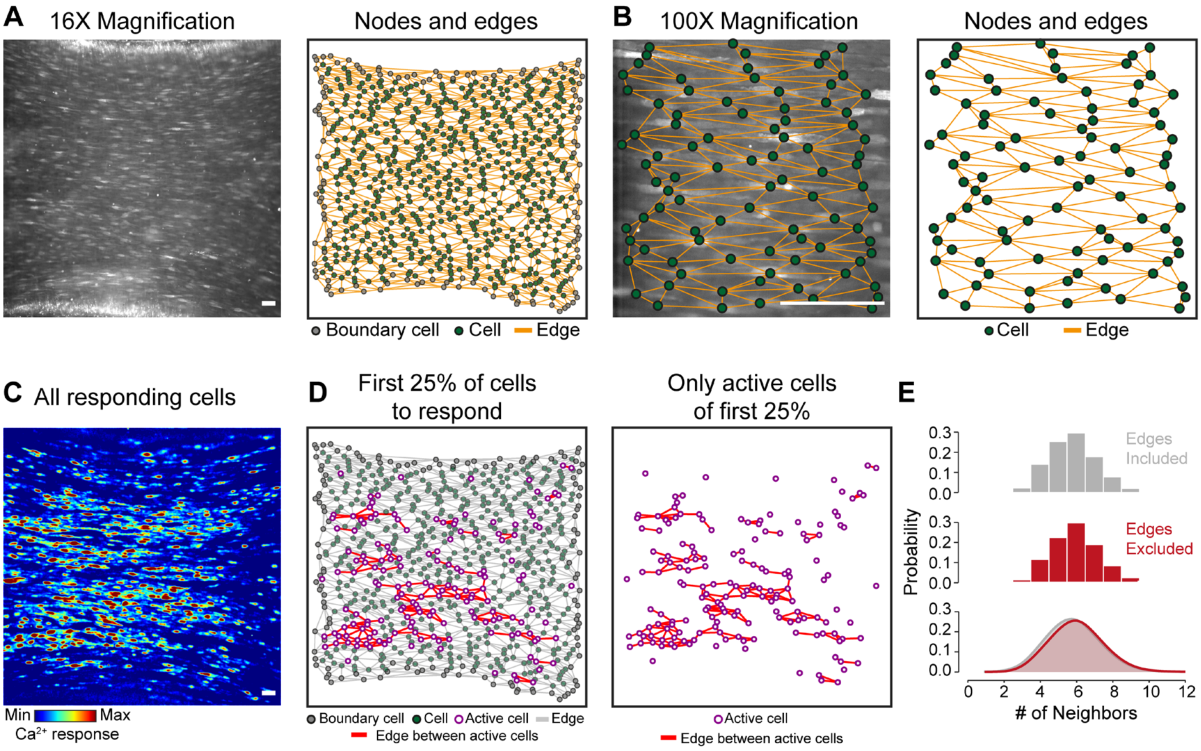

Supplementary Figure 2 – Neighbor analysis of endothelial cell activity.(A) High-resolution, low-magnification Ca^{2+} image (left) and corresponding structural network (right) of a large section of the endothelium of an intact second-order mesenteric artery. In the right panel, the centre of each cell is indicated by green ROIs, and connections (edges) between neighboring cells are shown as orange lines. Cells positioned at the boundary of the field-of-view are colored grey. (B) High-resolution, high-magnification image (left) of a region of endothelial cells from the same field of endothelial cells shown in A, and corresponding structural network (right). (C) Pseudo-colored Ca^{2+} image illustrating acetylcholine-evoked (ACh, 15 nM) Ca^{2+} activity throughout a 5-minute recording. (D) Location of ACh-responsive cells (first 25% of cells to respond, purple) shown with (left) and without (right) ACh-insensitive cells (green). (E) Probability density distributions showing the number of neighbors for each cell in A with boundary cells included (grey) or excluded (red). Scale bars, 50 µm.

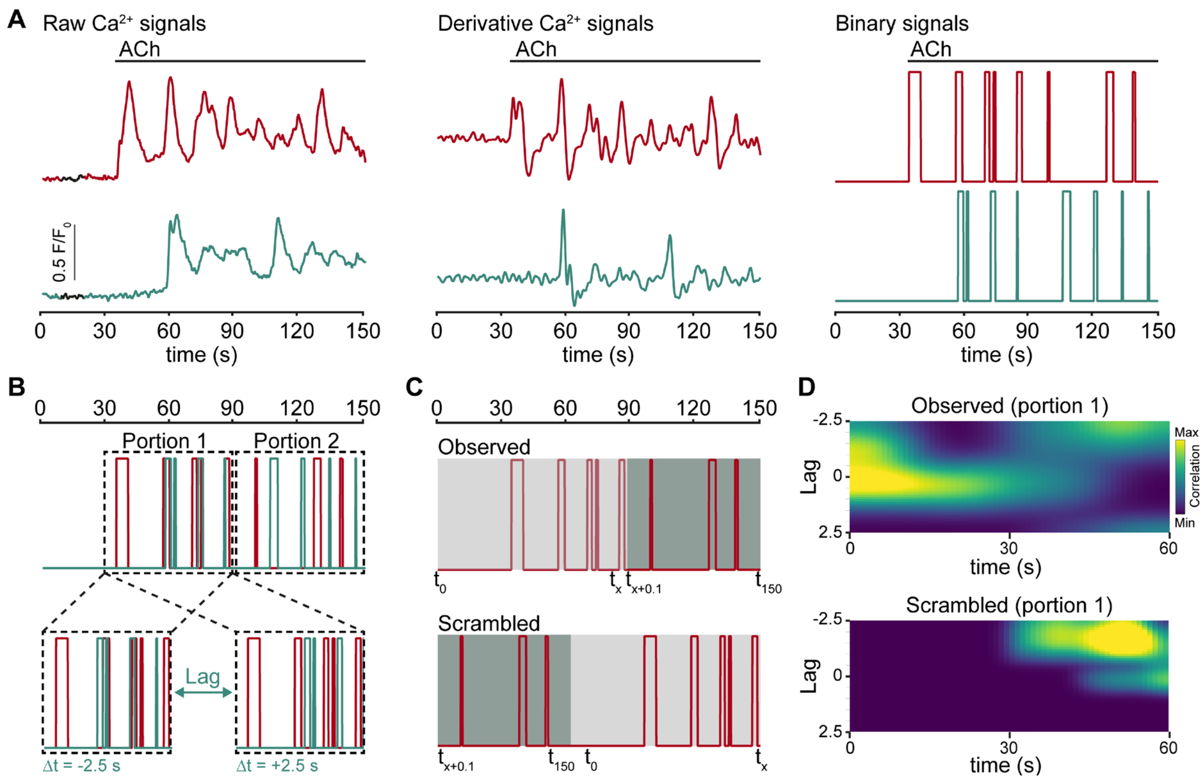

Supplementary Figure 3 – Cross-correlation analysis.

(A) Processing of Ca^{2+} signals for cross-correlation analysis. False correlations between Ca^{2+} signals may occur due to underlying trends. These were eliminated by taking the first derivative (middle) of the raw signal (left). Artefacts due to amplitude fluctuations and sparse data (zero values) were eliminated by digitising signal magnitudes (right) between zero (below threshold) and one (above threshold). Threshold was set as more than 5 standard deviations above noise. Here noise is defined as fluctuations in baseline signal over a 10 second period (black section). (B) Rolling window cross-correlation analysis. The rolling window cross-correlation between pairwise time series Ca^{2+} signals shifts one signal from -2.5 s to +2.5 s (lag = 2.5 s) and calculates the correlation at each 0.1 s interval. The maximal cross-correlation of two pairwise signals is calculated. (C) A scrambled dataset is generated by translating an observed signal at a random time point. (D) A correlogram of observed signals in A (top) and scrambled (bottom) generated from the red signal in C.

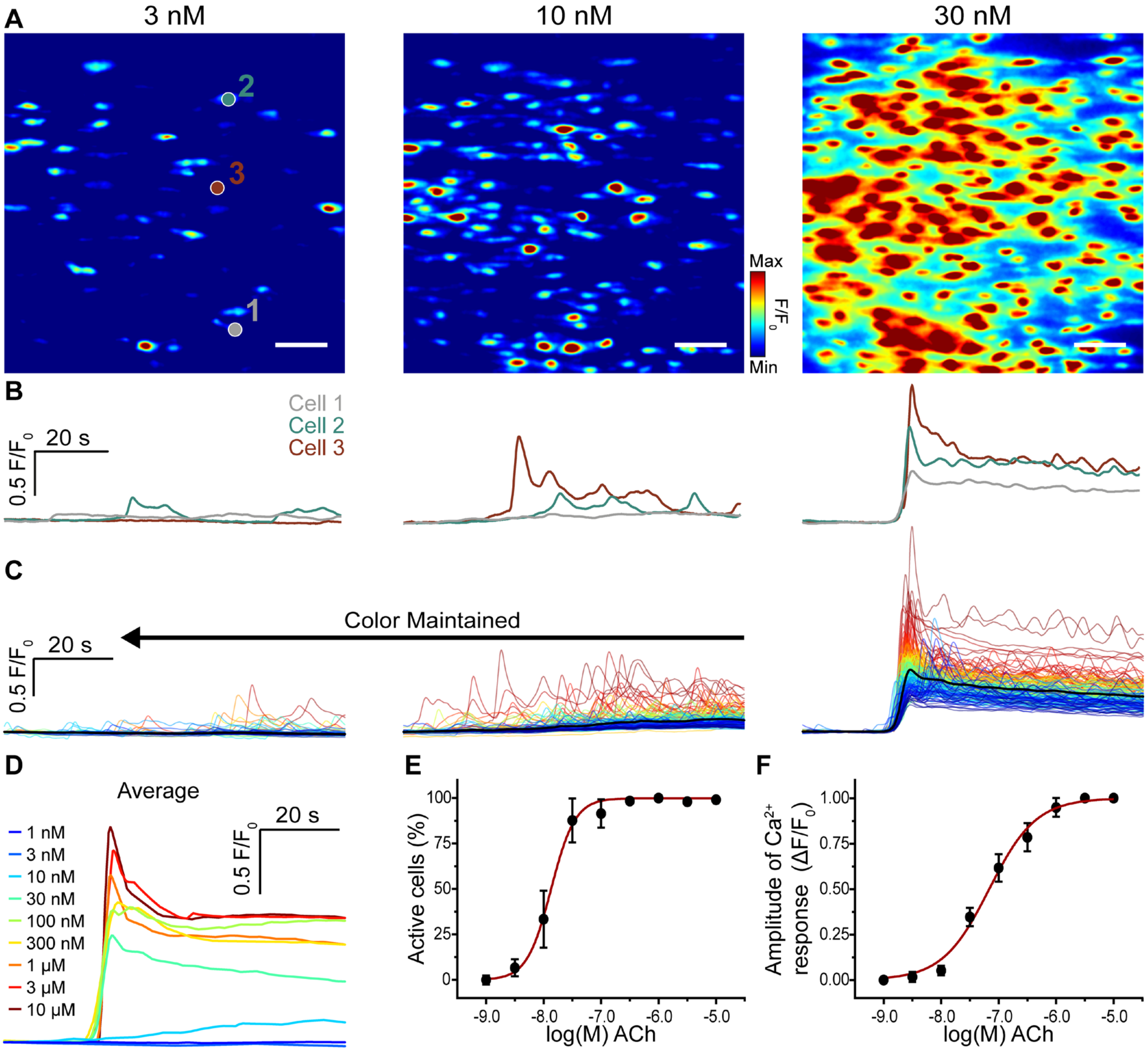

Supplementary Figure 4 – Concentration dependent ACh-evoked Ca^{2+} response.

(A) Representative composite image of Ca^{2+} activity in *en face* second order mesenteric artery endothelium to increasing concentrations of ACh. (B) Ca^{2+} traces from individual cells indicated in (A). (C) Ca^{2+} signals from all individual cells in (A) Colors were assigned to the traces based on the amplitude of the initial response to ACh in each cell. Red indicated the highest amplitude and blue the lowest amplitude. Colors were assigned to the highest ACh concentration (far right) first and maintained across the previous concentrations. Thus allowing for direct comparison of the same cells between different ACh concentrations. (D) Average Ca^{2+} response (F/F_0) from all cells for each ACh concentration. (E) Percentage of all cells active at each concentration of ACh. (F) Average activity (F/F_0) of all cells for each concentration of ACh. Data are representative of n = 5 independent experiments from different artery preparations from different animals. Scale Bars, 50 μm.

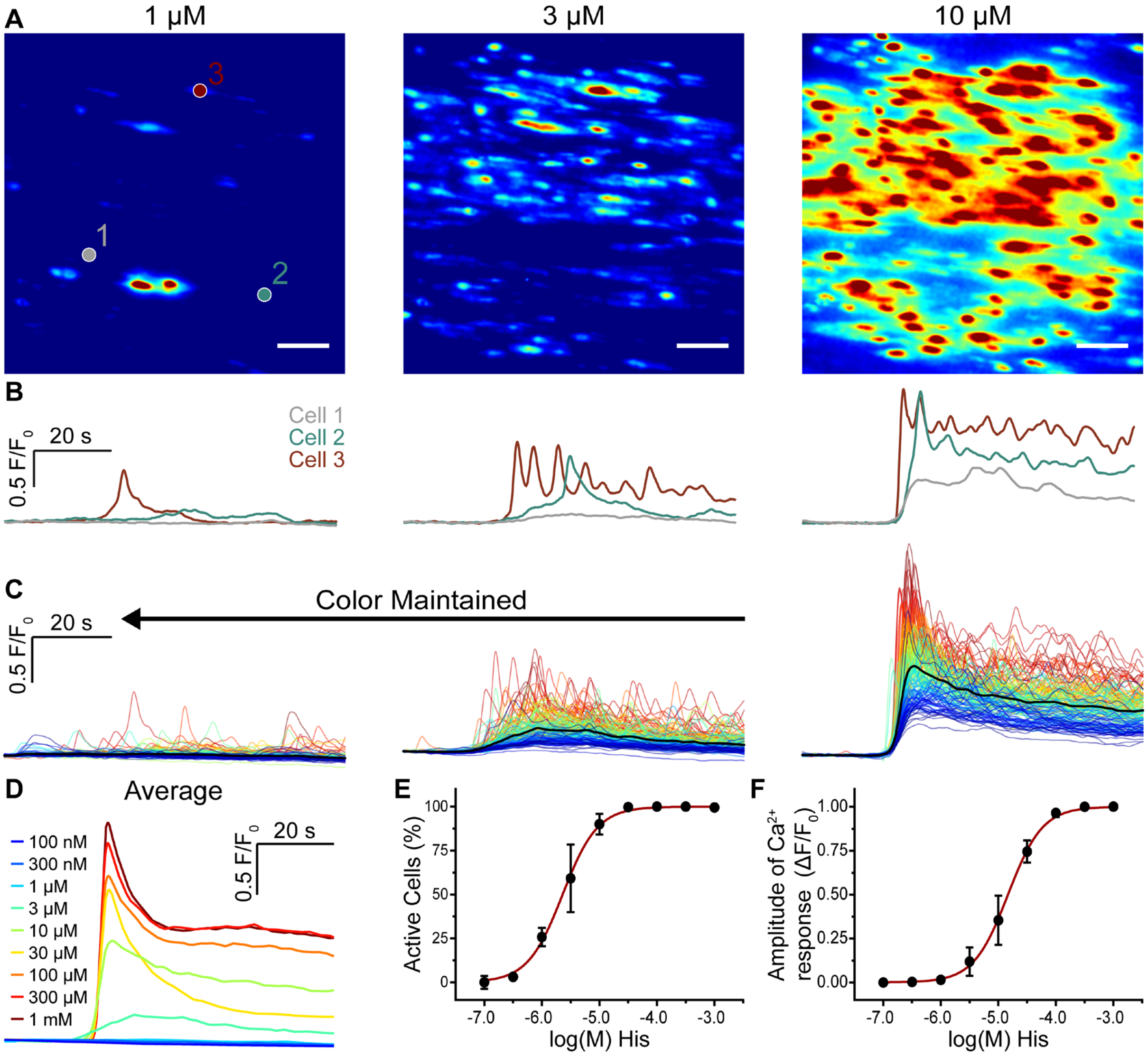

Supplementary Figure 5 - Concentration dependent histamine-evoked Ca^{2+} response.

(A) Representative composite image of Ca^{2+} activity in *en face* second order mesenteric artery endothelium to increasing concentrations of histamine. (B) Ca^{2+} traces from individual cells indicated in (A). (C) Ca^{2+} signals from all individual cells in (A) Colors were assigned to the traces based on the amplitude of the initial response to histamine in each cell. Red indicated the highest amplitude and blue the lowest amplitude. Colors were assigned to the highest histamine concentration (far right) first and maintained across the previous concentrations. Thus allowing for direct comparison of the same cells between different histamine concentrations. (D) Average Ca^{2+} response (F/F_0) from all cells for each histamine concentration. (E) Percentage of all cells active at each concentration of histamine. (F) Average activity (F/F_0) of all cells for each concentration of histamine. Data are representative of n = 5 independent experiments from different artery preparations from different animals. Scale Bars, 50 μm.

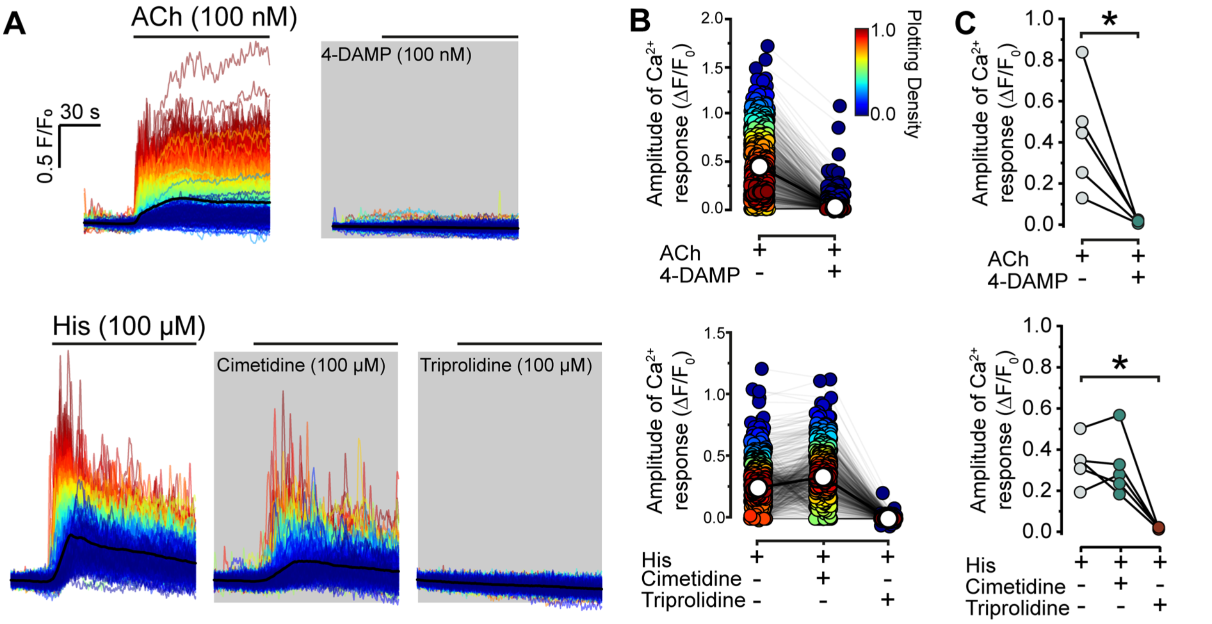

Supplementary Figure 6 – Receptor subtypes involved in muscarinic and histaminergic Ca^{2+} signaling in the mesenteric artery.

(A) Ca^{2+} signals from all individual cells (~1000 cells), before (left) and after incubation (5 min) with the indicated antagonist. All traces from individual cells are overlaid and colored based on amplitude of initial peak (highest amplitude in red, through to lowest in blue) and the black line represents the average. (B) Paired peak Ca^{2+} response ($\Delta F/F_0$) from individual cells before and after indicated treatment. Each circle represents an individual cell and these are matched for each treatment (grey lines) from a single experiment. The average response is marked by white circles and matched across treatments by a solid black line. The plotting density color coding indicates the distribution of peak $\Delta F/F_0$ values. Red indicates a higher frequency of occurrence of a particular peak $\Delta F/F_0$ value, and blue indicates a low frequency of occurrence of a peak $\Delta F/F_0$ value. (C) Summary data illustrating average Ca^{2+} response to acetylcholine (ACh) and histamine. Data are representative of n = 5 independent experiments, from artery preparations, from different animals; *$P < 0.05$, either paired Student t test or One-way ANOVA followed by Tukey's multiple comparisons test.

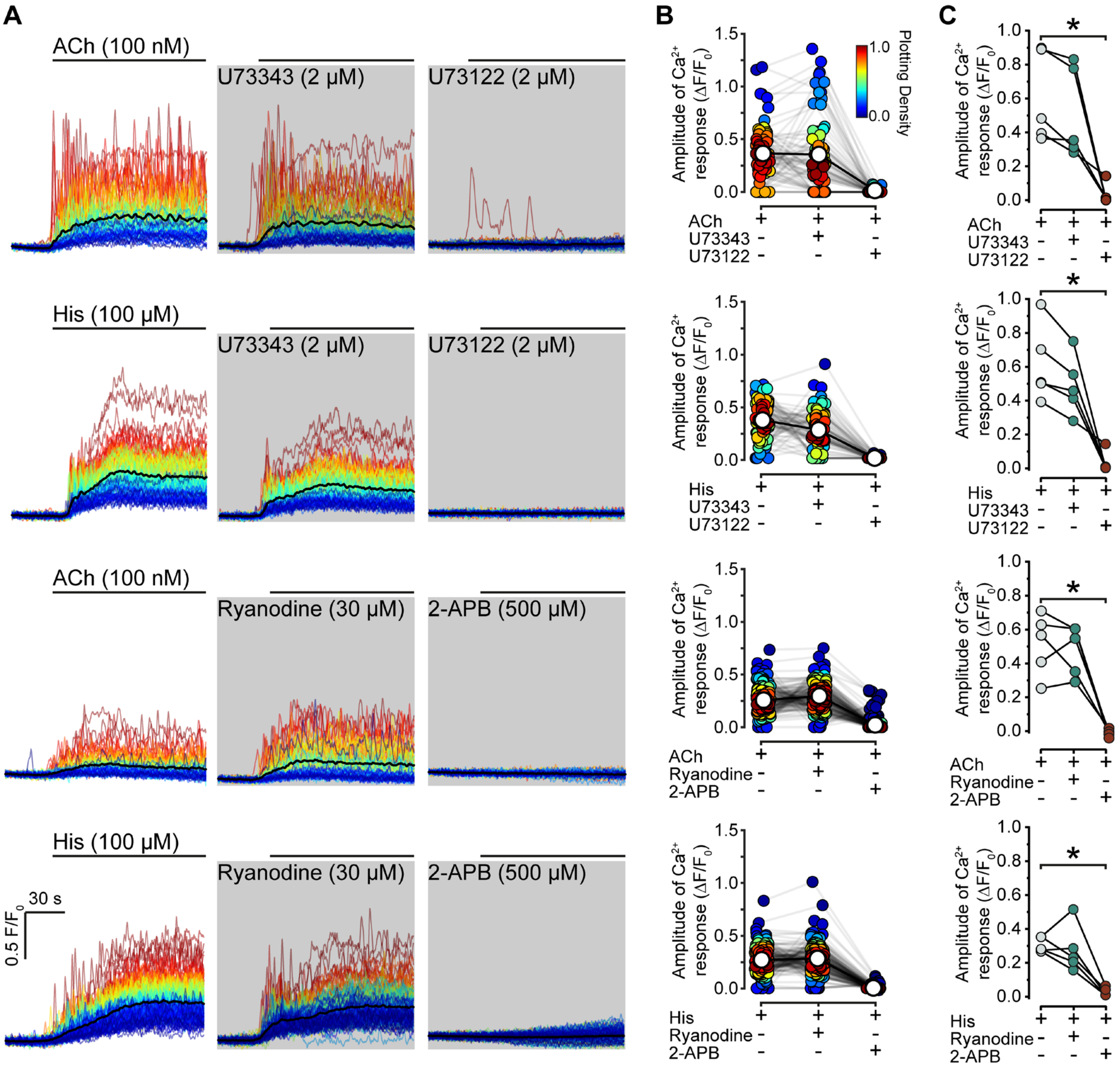

Supplementary Figure 7 – IP_3 controls muscarinic and histaminergic-mediated Ca^{2+} release in mesenteric endothelial cells.

(A) Ca^{2+} signals from all individual cells (~200 cells) showing the effects the Phospholipase C (PLC) inactive analogue, U73343 (2 µM, 10 min), the active form U73122 (2 µM, 10 min), the ryanodine receptor blocker, ryanodine (30 µM, 20 min) and the IP_3 receptor antagonist 2-aminoethoxydiphenyl borate, 2-APB (500 µM, 20 min) on muscarinic and histaminergic Ca^{2+} responses. All traces from individual cells are overlaid and colored based on amplitude of initial peak (highest amplitude in red, through to lowest in blue) and the black line represents the average. (B) Paired peak Ca^{2+} response ($\Delta F/F_0$) from individual cells before and after treatment with U73343, U73122, Ryanodine or 2-APB. Each circle represents an individual cell and these are matched for each treatment (grey lines) from a single experiment. The average response is marked by white circles and matched across treatments by a solid black line. The plotting density color coding indicates the distribution of peak $\Delta F/F_0$ values. Red indicates a higher frequency of occurrence of a particular peak $\Delta F/F_0$ value, and blue indicates a low frequency of occurrence of a peak $\Delta F/F_0$ value. (C) Summary data illustrating average Ca^{2+} response to acetylcholine (ACh) and histamine before and after treatment. Data are representative of n = 5 independent experiments, from artery preparations, from different animals; *$P < 0.05$, One-way ANOVA followed by Tukey's multiple comparisons test.

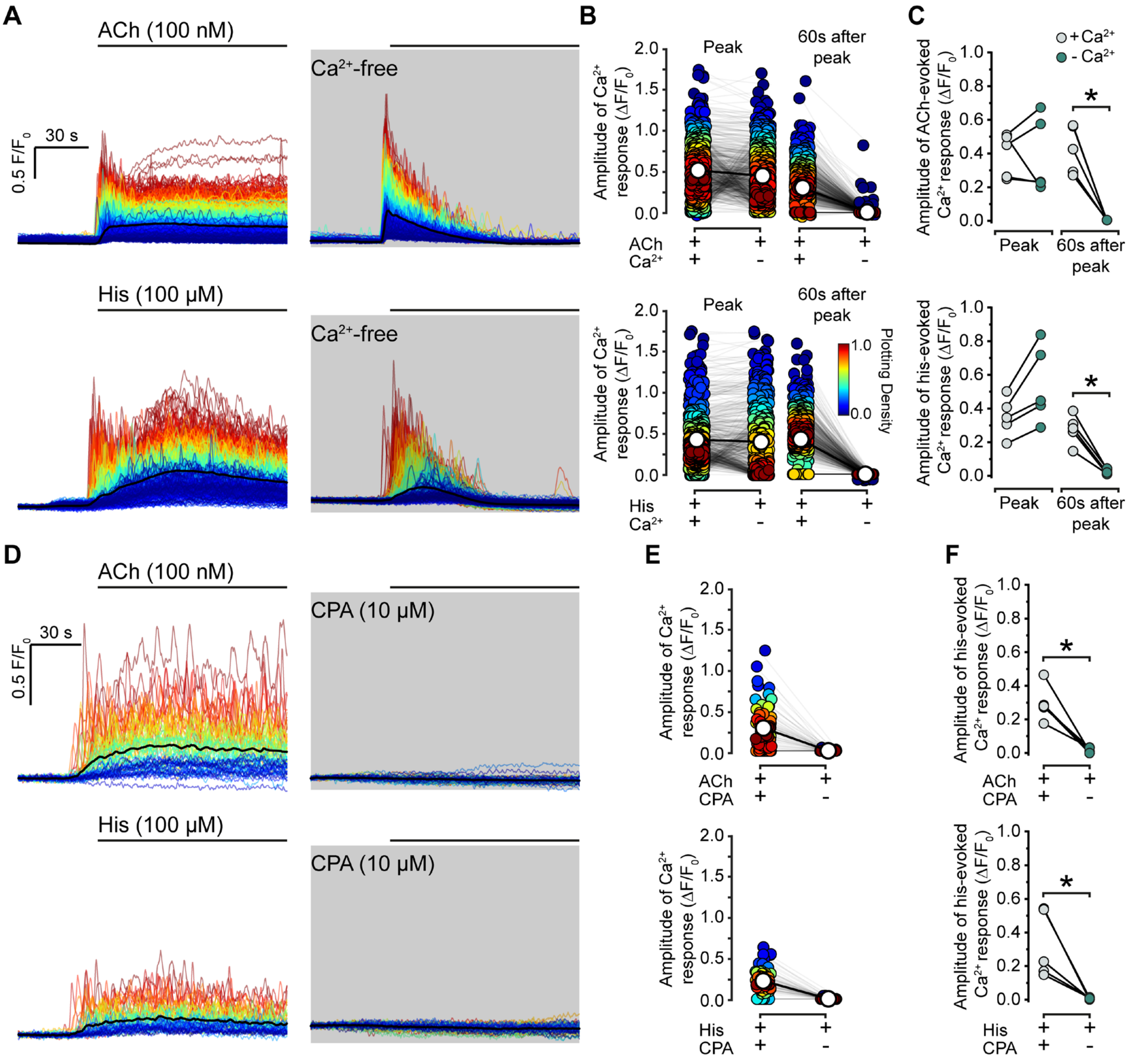

Supplementary Figure 8 – Effect of Ca^{2+} influx and internal Store depletion on muscarinic and histaminergic Ca^{2+} signaling.

(A) Ca^{2+} signals from all individual cells showing the effects of Ca^{2+} influx (Ca^{2+}-free PSS) and store depletion with cyclopiazonic acid (CPA; 10 µM, 5 min) on muscarinic and histaminergic evoked Ca^{2+} responses. Traces from individual cells are overlaid and colored based on amplitude of initial peak (highest amplitude in red, through to lowest in blue) and the black line represents the average. (B) Paired peak Ca^{2+} response ($\Delta F/F_0$) from individual cells before and after treatment. Each circle represents an individual cell and these are matched for each treatment (grey lines) from a single experiment. The plotting density color coding indicates the distribution of peak $\Delta F/F_0$ values. Red indicates a higher frequency of occurrence of a particular peak $\Delta F/F_0$ value, and blue indicates a low frequency of occurrence of a peak $\Delta F/F_0$ value. (C) Summary data illustrating average Ca^{2+} response to acetylcholine (Ach) and histamine before and after treatment. Data are representative of n = 5 independent experiments, from artery preparations, from different animals; *$P < 0.05$, paired Student t test.

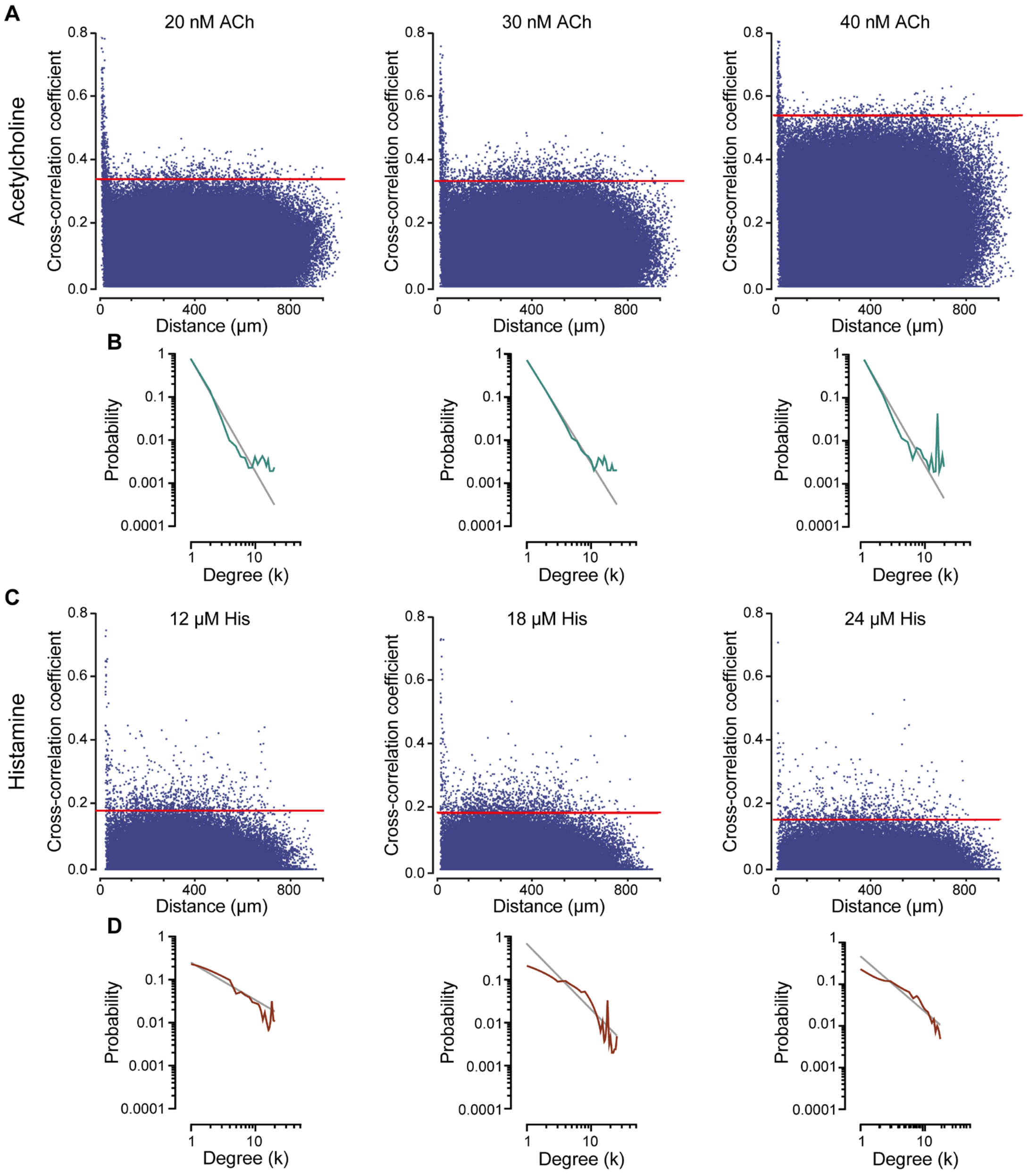

Supplementary Figure 9 – Network analysis of concentration-dependence of acetylcholine- and histamine-evoked Ca^{2+} responses in the endothelium.

(A) Scatterplot of cross-correlation coefficients plotted as a function of intercellular distance and (B) probability distribution on a log-log scale with a linear regression fit in grey for increasing concentrations of acetylcholine. (C) Scatterplot of cross-correlation coefficients plotted as a function of intercellular distance and (D) probability distribution on a log-log scale with a linear regression fit in grey for increasing concentrations of histamine.

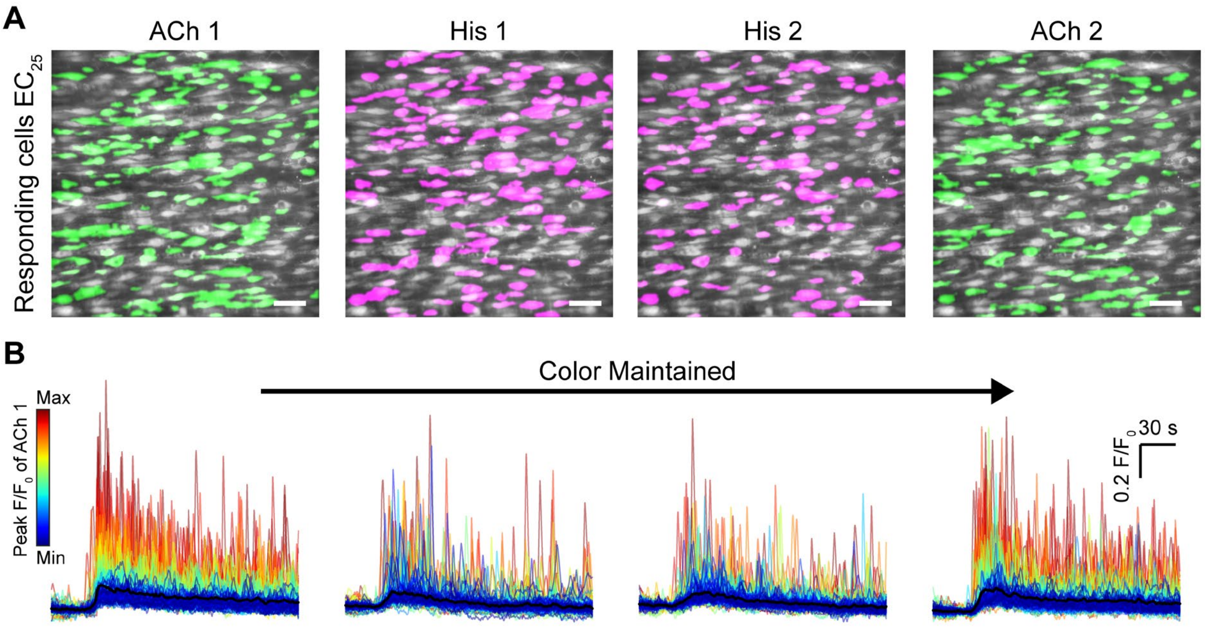

Supplementary Figure 10 - Reproducibility of endothelial cell Ca^{2+} response.

(A) Representative composite image of Ca^{2+} activity in *en face* second order mesenteric artery endothelium to the EC_{25} of acetylcholine (ACh, green) and histamine (magenta). (B) Ca^{2+} signals from all individual cells in (A) Colors were assigned to the traces based on the amplitude of the response to the first ACh application in each cell and maintained across all subsequent histamine or ACh applications. Red indicated the highest amplitude and blue the lowest amplitude. Scale bars, 50 µm.

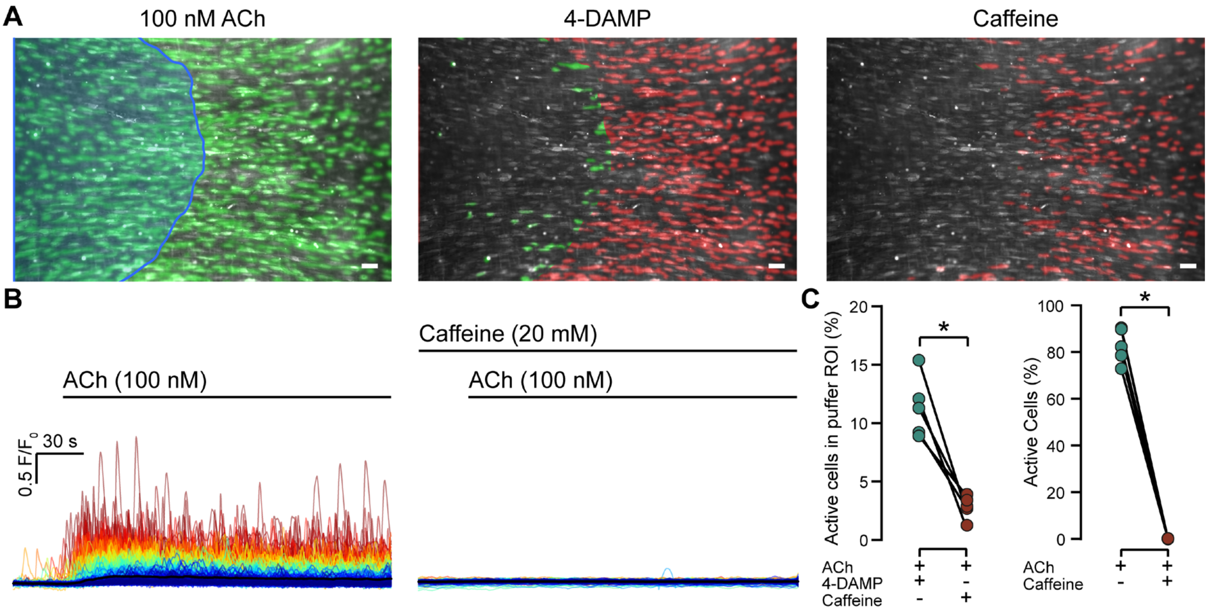

Supplementary Figure 11 - Endothelial cell communication in intact mesenteric arteries.

(A) Representative composite image of Ca^{2+} activity in *en face* second order mesenteric artery endothelium to acetylcholine (ACh, green) applied to the entire preparation. *Left* The puffer region is highlighted by the blue line. The M3 receptor blocker, 4-DAMP (100 nM; *middle*), and IP_3R blocker caffeine (20 mM; *right*), were applied by pressure ejection from a puffer pipette, against a stream of PSS flow, to the region highlighted by the blue line in the left-panel. The fluorescent dye Sulforhodamine B (2µM) was included in the puffer pipette solution in each case to identify the cells blocked by the antagonists. Active cells in the blocked region are highlighted in green and active cells outside the blocked region are highlighted in red (B) Ca^{2+} signals from all individual cells in (A) Colors were assigned to the traces based on the amplitude of the response to the first ACh application in each cell and maintained across all subsequent ACh applications. Red indicated the highest amplitude and blue the lowest amplitude. (C) Summary data plotting the percentage of active cells in the puffer region (Left) and all cells before and after global caffeine application across the entire endothelium (right). Data are representative of n = 5 independent experiments, from artery preparations, from different animals; *P < 0.05, paired Student t test. Scale bars, 50 µm.

SUPPLEMENTARY VIDEOS

Supplementary Video 1. Acetylcholine Concentration Response. Concentration-dependent acetylcholine-evoked endothelial Ca^{2+} responses. Top panel, representative videos of acetylcholine (ACh)-evoked (3 nM, 10 nM and 30 nM) endothelial Ca^{2+} activity with the raw traces (left) and fractional change in fluorescence (F/F_0; right) shown. Middle panel, Ca^{2+} signals from 3 randomly selected cells from the population at each (3 nM, 10 nM and 30 nM) concentration. The Ca^{2+} signals from same cells are shown at each concentration. Lower panel, heat map of Ca^{2+} activity for all cells in the field of view (172 cells) at each concentration (3 nM, 10 nM and 30 nM). Each line of the heat map represents an individual cell. Arteries were opened and pinned flat before endothelial cells were preferentially labelled with the fluorescent Ca^{2+} indicator, Cal-520/AM (5 µM), and visualized using high-resolution wide field single-photon microscopy. The dataset in the video is summarized in Supplementary Figure 4. Scale bars, 50 µm.

Supplementary Video 2. Histamine Concentration Response. Concentration-dependent histamine-evoked endothelial Ca^{2+} responses. Top panel, representative videos of histamine (His)-evoked (1 µM, 3 µM and 10 µM) endothelial Ca^{2+} activity with the raw traces (left) and fractional change in fluorescence (F/F_0; right) shown. Middle panel, Ca^{2+} signals from 3 randomly selected cells from the population at each (1 µM, 3 µM and 10 µM) concentration. The Ca^{2+} signals from same cells are shown at each concentration. Lower panel, heat map of Ca^{2+} activity for all cells in the field of view (152 cells) at each concentration (1 µM, 3 µM and 10 µM). Each line of the heat map represents an individual cell. Arteries were opened and pinned flat before endothelial cells were preferentially labelled with the fluorescent Ca^{2+} indicator, Cal-520/AM (5 µM), and visualized using high-resolution wide field single-photon microscopy. The dataset in the video is summarized in Supplementary Figure 5. Scale bars, 50 µm.

Supplementary Video 3. Spatially distinct cell clusters activated by the EC_{25} concentrations of acetylcholine and histamine. The upper panels show the spatially-distinct clusters of cells that respond to the EC_{25} of acetylcholine (green; left) and histamine (magenta; right) in the same preparation. The lower panel shows an overlay of the responses to acetylcholine (green) and histamine (magenta) indicating the separate populations of cells activated by each agonist. Scale bars, 50 µm.

Supplementary Video 4. Short-cut activation of cells at distance from the activation site. Raw fluorescence (left panel), F/F_0 (middle panel) and cell activation time (right panel) after photorelease of caged IP_3. After local activation by photoreleased caged IP_3 (white circle, uncaging area), Ca^{2+} signals moved outwards from the photolysis site at an approximately constant speed. However, some cells positioned at distance from the photolysis site were activated before cells positioned closer to the photolysis site, giving a faster propagation velocity and the impression of signals 'skipping' ahead of the wave front.
